# Researcher Perceptions of Inclusion of Study Participants Who Use Languages Other Than English

**DOI:** 10.1001/jamanetworkopen.2025.2380

**Published:** 2025-03-28

**Authors:** Henry Hoffman, Tran T. Doan, Olivia Migliori, Alisa Khan, Jaime Sidani, Sabrina Liu, Abby Jo Perez, Lani Mears, Benoit Kihumbu, Khara Timsina, Diego Chaves-Gnecco, John D. Cowden, Maya I. Ragavan

**Affiliations:** 1 University of Pittsburgh School of Medicine, Pittsburgh, Pennsylvania; 2 Department of Health Systems, Management, and Policy, Colorado School of Public Health, Aurora; 3 Division of General Academic Pediatrics, Department of Pediatrics, University of Pittsburgh, Pittsburgh, Pennsylvania; 4 Division of General Pediatrics, Department of Pediatrics, Boston Children’s Hospital, Boston, Massachusetts; 5 Department of Pediatrics, Harvard Medical School, Boston, Massachusetts; 6 Department of Behavioral and Community Health Sciences, University of Pittsburgh School of Public Health, Pittsburgh, Pennsylvania; 7 Asian Pacific American Labor Alliance-Pittsburgh, Pittsburgh, Pennsylvania; 8 Hello Neighbor, Pittsburgh, Pennsylvania; 9 Filipino American Association of Pittsburgh, Pittsburgh, Pennsylvania; 10 Congolese Union of Pittsburgh, Pittsburgh, Pennsylvania; 11 Bhutanese Community Association of Pittsburgh, Pittsburgh, Pennsylvania; 12 Division of General Academic Pediatrics, Department of Pediatrics, Children’s Mercy Kansas City, Kansas City, Missouri; 13 Department of Pediatrics, University of Pittsburgh Medical Center Children’s Hospital of Pittsburgh, Pittsburgh, Pennsylvania

## Abstract

**Importance:**

Despite constituting 8% of the US population, people who speak languages other than English (LOE) are consistently underrepresented in health-focused research. To make research more inclusive and generalizable, it is crucial to better understand researcher perspectives on barriers to inclusion of participants using LOE and solutions to promote language justice.

**Objective:**

To assess researcher perspectives on barriers to and best practices for inclusion of participants using LOE and to generate strategies to make research more inclusive.

**Design, Setting, and Participants:**

This exploratory cross-sectional survey study used an anonymous digital Qualtrics survey distributed between March 1 and June 30, 2023. The study was conducted among principal investigators (PIs) and research coordinators at the University of Pittsburgh, Pennsylvania. Any PIs or research coordinators who conducted human participant research in the past 5 years were eligible to participate. A convenience sample, using department emails, research electronic mailing lists, a recruitment repository, and word of mouth, was recruited.

**Main Outcomes and Measures:**

Survey questions, drafted with input from community partners, assessed researcher demographics, experience working with participants using LOE, barriers to inclusion, and recommendations to increase research inclusivity. All variables were analyzed using descriptive statistics to calculate means, SDs, and frequencies.

**Results:**

There were 339 respondents who completed the survey (260 cisgender females or women [76.7%]). Of these respondents, 127 (37.5%) were PIs and 212 (62.5%) were research coordinators. In terms of race and ethnicity, 8.8% were Asian; 3.8% were Black, African, or African American; 2.4% were Hispanic, Latino, Latina, Latine, or Latinx; 0.9% were Middle Eastern or North African; 70.5% were White; 6.5% were of other race or ethnicity or were multiracial; 2.7% preferred not to say; and 4.4% had missing data. Most respondents primarily conducted clinical research (239 [70.5%]), and 170 (50.1%) worked with participants using LOE in the prior 5 years. In 188 reported cases in which inclusion occurred, 78 respondents (41.5%) did not proactively include participants using LOE but instead reactively included interested participants after studies had begun. Respondents listed lack of training, time and scheduling challenges around interpreter services, and budget constraints as barriers to inclusion. Recommendations to improve inclusion were made by 272 respondents (80.2%), 265 (78.2%) of whom suggested access to low-cost interpreters and translators, 249 (73.5%) of whom suggested training, and 272 (80.2%) of whom suggested availability of validated measures in different languages.

**Conclusions and Relevance:**

In this survey study of health PIs and research coordinators affiliated with 1 academic institution, respondents faced multiple barriers to including participants who use LOE in their studies. Because a lack of language representation may compromise the quality and applicability of research, purposeful individual and institutional investments are needed to overcome these barriers.

## Key Points

**Question:**

What do researchers perceive as barriers to and best practices for inclusion of study participants who use languages other than English (LOE)?

**Findings:**

In this survey study that included 339 researchers, 50% reported working with participants using LOE at least once in the prior 5 years. To promote inclusivity of participants who use LOE, respondents recommended increased access to interpretation and translation services, guidance on budgeting, methods training, and networking with other researchers.

**Meaning:**

The findings of this study suggest that multilevel solutions, including institutional support, are needed to increase inclusion of participants who use LOE and to promote more inclusive, generalizable research.

## Teaser

This survey study evaluates researchers’ perceptions of barriers to and best practices for inclusion of study participants who use languages other than English.

## Introduction

People who use languages other than English (LOE) constitute 8.2% of the total US population and disproportionately experience health-related disparities.^1^ Research has shown that patients speaking LOE may experience higher rates of hospital adverse medical events,^2^ more frequent medication administration errors,^3^ higher rates of readmissions,^4^ longer average hospital stays,^5^ and higher overall mortality.^6^ One strategy to address these disparities is to ensure research representation for individuals who use LOE. People using LOE view research participation as a valuable way to improve the health and well-being of their communities.^7^ Moreover, the inclusion of diverse communities enhances research quality and generalizability and has been shown to improve health outcomes and disrupt health disparities.^8,9,10,11^ However, individuals using LOE are frequently excluded from health research.^12,13,14,15,16,17^ For example, a systematic review of pediatric literature published between 2012 and 2021 found that only 9% of articles included participants using LOE.^12^ Even when participants using LOE are included in research, their inclusion is often suboptimal. A qualitative study interviewing community members using LOE about their perspectives on research found that participants were often asked to complete research in English and thus did not fully understand the activities included in their research participation.^7^ Both exclusion and improper inclusion are problematic and must be better understood to improve equity and quality of research.

Mistrust is frequently described as a fundamental barrier to participation in research among groups experiencing structural marginalization, such as racial and ethnic minoritized groups, people identifying as sexually or gender diverse, and people living with disabilities.^18,19,20^ Recent work described similar mistrust among individuals who use LOE.^7^ This mistrust stems from a well-documented history of researchers conducting unethical studies causing harm to some individuals in these communities.^21^ Given this history, it is vital that the responsibility of making research more inclusive shift from participants who use LOE to researchers and research institutions.^22^ However, descriptions of researchers’ attitudes and recommendations regarding inclusion of participants who use LOE are lacking. Therefore, in this exploratory cross-sectional survey study, we examined the perspectives of researchers on the inclusion of participants who use LOE in health-focused human participant research. Our objectives were to assess how frequently researchers include participants who use LOE in their research, identify barriers to such inclusion, and generate strategies to increase the accessibility and trustworthiness of research among individuals who use LOE.

## Methods

### Overview

We conducted a cross-sectional survey study of researchers at the University of Pittsburgh, Pennsylvania. The University of Pittsburgh has several language resources available for researchers, including guidelines from the institutional review board (IRB), a responsible conduct in research session on multilingual methods, language equity consults available through the University of Pittsburgh Clinical and Translational Science Institute, and contracting support with a translation company. We chose a cross-sectional survey design^23^ because, to our knowledge, there currently are insufficient timely or relevant existing data on the topic. The University of Pittsburgh IRB deemed this study exempt from review because it was not considered human participant research. The IRB requested an information prescription be provided to each survey respondent rather than obtaining written or oral consent. We followed the American Association for Public Opinion Research (AAPOR) reporting guideline.

The study was conducted in collaboration with the Immigrant and Refugee Equity Project, a community-academic collaboration comprising immigrant-serving and refugee-serving organizations; health care clinicians; and researchers from medicine, public health, social work, immersive media, and linguistics, which meets monthly to discuss research projects related to language equity and immigrant and refugee health. Immigrant and Refugee Equity Project members (H.H., O.M., J.S., S.L., A.J.P., L.M., B.K., K.T., D.C.-G., and M.I.R.) reviewed the survey, provided insight on data interpretation, and helped coauthor the manuscript. Data collection occurred from March 1 to June 30, 2023.

### Respondents and Recruitment

Eligible respondents were defined as any principal investigator (PI), including graduate students, or research coordinator currently affiliated with the University of Pittsburgh who had led a human participant research project in the prior 5 years. Research coordinators were included because they often have intimate knowledge of recruitment, data collection, and other aspects of study design.^24^ A convenience, nonprobability-based sample was recruited using department emails, electronic mailing lists from the University of Pittsburgh Clinical and Translational Science Institute, an online recruitment repository, and word of mouth. Respondents self-reported their race and ethnicity, which included Asian; Black, African, or African American; Hispanic, Latino, Latina, Latine, or Latinx; Middle Eastern or North African; White; other race or ethnicity or multiracial; preferred not to say; and missing data. Race and ethnicity were included in the study because we recognize that race and ethnicity are social constructs, and thus we asked about racial and ethnic identity to better understand the intersectional identities and experiences of respondents.

### Survey Development and Measures

We created a new survey for this study because we found no validated measure to assess our research questions in the literature (eAppendices 1 and 2 in Supplement 1). Two team members (O.M. and M.I.R.) drafted all questions, and iterative changes were made based on feedback from the Immigrant and Refugee Equity Project. All questions were aligned with the AAPOR best practices guidelines in that they were short, simple, free of bias language, logically ordered, either close-ended or open-ended, and asked about only 1 concept at a time. The survey was then pilot tested with 7 PIs and research coordinators, with their feedback incorporated in the final survey.

The survey domains included (1) demographics (eg, gender identity, race and ethnicity, primary role in the university, and LOE spoken with at least basic proficiency); (2) inclusion of participants who use LOE in research and in how many studies (answers were all, most but not all, about half, some but less than half, or none); (3) barriers to multilingual research in the domains of language, training, and budgeting (answers were based on a 5-point Likert scale from 1 [strongly agree] to 5 [strongly disagree], except for budgeting questions, which were answered as true, false, or unknown for respondents not actively involved in budgeting); and (4) recommendations for increasing inclusion of participants who use LOE in the domains of interpretation and translation, funding and budgeting, and training and institutional support (respondents could select multiple options among a list of recommendations). We also asked an open-ended question about additional tools needed to improve inclusion on individual and institutional levels. Of note, our survey used the term *limited English proficiency* instead of LOE, which is the term used in this article. We opted to use the former because it was the more recognized term at the time of survey administration, but we use *participants who use LOE* in this manuscript to avoid deficit-focused language.^25^

### Statistical Analysis

Respondents completed the 25-minute, 70-question survey electronically through Qualtrics and were provided an information script prior to starting the survey. Respondents were compensated $40. Data were monitored weekly to check for bots or other concerns. We cleaned the dataset by removing incomplete survey responses (any survey with >50% missing data) and missing data of those excluded from analysis. Likert scale responses (5 points) were aggregated into agree, neutral, and disagree. All variables were analyzed using descriptive statistics to calculate means, SDs, and frequencies. Data associated with community partnership across all domains were analyzed and presented separately due to the importance of this topic for research inclusivity. Free text responses were uploaded into the Dedoose qualitative software package, version 9.2.22 (SocioCultural Research Consultants LLC) and coded by 2 team members (H.H. and Callie Laubacher, BA) who met weekly to resolve any discrepancies. Quantitative analyses were conducted using Excel (Microsoft Corp). Thematic analysis was used to categorize codes into larger themes.^26^

## Results

### Respondent Characteristics

A total of 339 researchers participated (260 cisgender females or women [76.7%], 45 cisgender males or men [13.3%], 12 genderqueer or nonbinary [3.5%], 8 none of these gender identities [2.4%], and 14 missing data [4.1%]). Of these respondents, 127 (37.5%) were PIs and 212 (62.5%) were research coordinators. Due to use of convenience sampling, we were unable to calculate a response rate. Of the respondents, 30 were Asian (8.8%); 13 were Black, African, or African American (3.8%); 8 were Hispanic, Latino, Latina, Latine, or Latinx (2.4%); 3 were Middle Eastern or North African (0.9%); 239 were White (70.5%); 22 were of other race or ethnicity or were multiracial (6.5%); 9 preferred not to say (2.7%); and 15 had missing data (4.4%). Most respondents identified as White and as cisgender female. Clinical research was the type of human participant research most frequently reported by respondents (239 [70.5%]). A total of 212 respondents (62.5%) reported at least a basic proficiency in a non-English language; respondents reported using 41 languages total. Of all languages other than English used by respondents (329 total instances), 217 respondents (66.0%) were basic or fairly proficient, and 112 (34.0%) had a good, advanced, or fluent proficiency. Respondent characteristics are presented in Table 1.

**Table 1.** Demographic Characteristics of Researcher Respondents

Characteristic	Respondents, No. (%) (N = 339)
Response to prompt “Has someone with limited English proficiency participated in one of your human participant studies in the last 5 years?”	
No	169 (49.9)
Yes	170 (50.1)
Primary role	
Principal investigator	127 (37.5)
Project coordinator	212 (62.5)
Experience in primary role, y	
<1	39 (11.5)
1-3	80 (23.6)
4-6	56 (16.5)
7-10	43 (12.7)
11-15	53 (15.6)
16-20	23 (6.8)
>20	45 (13.3)
Race and ethnicity	
Asian	30 (8.8)
Black, African, or African American	13 (3.8)
Hispanic, Latino, Latina, Latine, or Latinx	8 (2.4)
Middle Eastern or North African	3 (0.9)
White	239 (70.5)
Other or multiracial	22 (6.5)
Prefer not to say	9 (2.7)
Missing	15 (4.4)
Gender identity	
Cisgender female or woman	260 (76.7)
Cisgender male or man	45 (13.3)
Genderqueer or nonbinary	12 (3.5)
None	8 (2.4)
Missing	14 (4.1)
Immigration generation	
1st	48 (14.2)
2nd	37 (10.9)
≥3rd	66 (19.5)
None	175 (51.6)
Missing	13 (3.8)
Primary type of research conducted^a^	
Basic science	52 (15.4)
Clinical	239 (70.5)
Population health	61 (18.0)
Community-partnered	60 (17.7)
Health services	69 (20.3)
Non–health-related human participant research	23 (6.8)
Other	7 (2.1)
School affiliation	
Arts and sciences	11 (3.2)
Dental medicine	4 (1.2)
Health and rehabilitation	8 (2.4)
Medicine	98 (28.9)
Nursing	6 (1.8)
Public health	23 (6.8)
Social work	5 (1.5)
Other	29 (8.6)
Missing	155 (45.7)
Reports at least basic LOE proficiency	
No	105 (31.0)
Yes	212 (62.5)
Missing	22 (6.5)
Proficiency level in reported LOE^b^	
Basic or fair	217 (66.0)
Good, advanced, or native	112 (34.0)

Abbreviation: LOE, languages other than English.

^a^ Respondents could select multiple types of research; thus, totals exceed 339 (100%).

^b^ Respondents could select multiple LOE that they spoke; thus, totals are reflective of reported instances of speaking LOE rather than the total number of respondents speaking LOE (eg, for a respondent who speaks Spanish and Portuguese, each language is reported separately as a discrete instance of LOE proficiency).

### Prior Experience Working With Participants Using LOE

Of 339 survey respondents, 170 (50.1%) stated that they had worked with participants using LOE in at least 1 human participant study in the prior 5 years. Of those, 108 (63.5%) included participants using LOE in less than half of their research studies. In 188 reported cases in which inclusion occurred, 88 respondents (46.8%) proactively included participants who use LOE (eg, from the start), 78 (41.5%) did not initially intend to include participants who use LOE but subsequently modified study methods based on interest in study participation during recruitment, and 22 respondents (11.7%) included participants in another way.

### Barriers to Inclusion

Respondents selected barriers along 3 domains: (1) recruitment, training, and staffing; (2) interpretation and translation services; and (3) budgeting and funding. Under recruitment, training, and staffing, the commonly reported barriers were a lack of training in how to include participants who use LOE (92 of 127 PIs only [72.4%]); studies focusing on an English-speaking population (198 of 339 respondents [58.4%]); mentors not including people who use LOE (71 of 127 PIs only [55.9%]); and team members speaking only English (183 of 339 respondents [54.0%]). Regarding interpretation and translation, among the 339 respondents, the most agreed-upon barriers were scheduling concerns when using interpreters (209 [61.7%]) and not knowing where to find professional interpretation and translation services (164 [48.4%]). Finally, 189 of the 339 respondents (55.8%) reported that they have not budgeted for language services in grant proposals. Selected barriers to inclusion are presented in Table 2 and Table 3.

**Table 2.** Perceived Barriers to Inclusion

Prompt	Researcher response, No. (%) (N = 339)			
	Agree	Neutral	Disagree	Missing data
**Recruitment, training, and staffing**				
My studies are focused on an English-speaking population.	198 (58.4)	49 (14.5)	89 (26.3)	3 (0.9)
I have never had someone with limited English proficiency interested in any of my research studies.	93 (27.4)	64 (18.9)	179 (52.8)	3 (0.9)
My study team members only speak English.	183 (54.0)	40 (11.8)	112 (33.0)	4 (1.2)
I haven’t tried to hire bilingual/multilingual study team members.	104 (30.7)	120 (35.4)	110 (32.4)	5 (1.5)
I don’t know where to find a community-based organization who works with limited English-proficient communities.^a^	48 (37.8)	25 (19.7)	51 (40.2)	3 (2.4)
I was not trained to include people with limited English proficiency into my research.^a^	92 (72.4)	11 (8.7)	22 (17.3)	2 (1.6)
My mentors have not included people with limited English proficiency in their research.^a^	71 (55.9)	18 (14.2)	36 (28.3)	2 (1.6)
**Interpretation and translation services**				
I don’t know where to start looking for professional language services.	164 (48.4)	50 (14.7)	121 (35.7)	4 (1.2)
I am not familiar with using professional interpretation or translation services.	159 (46.9)	40 (11.8)	136 (40.1)	4 (1.2)
I anticipate time/scheduling barriers when utilizing interpretation/translation services.	209 (61.7)	69 (20.4)	57 (16.8)	4 (1.2)
I am concerned about the quality of professional interpretation or translation services.	138 (40.7)	72 (21.2)	125 (36.9)	4 (1.2)

^a^ Choices were given only to principal investigators.

**Table 3.** Budgeting and Funding Barriers

Budgeting and funding prompt	Researcher response, No. (%) (N = 339)			
	True	False	Unknown	Missing data
I haven’t budgeted for language services in grant proposals.	189 (55.8)	50 (14.7)	96 (28.3)	4 (1.2)
I budgeted for language services in grant proposals that were not funded.	22 (6.5)	176 (51.9)	137 (40.4)	4 (1.2)
I wanted to include participants with limited English proficiency but it was too costly.	52 (15.3)	141 (41.6)	142 (41.9)	4 (1.2)
I wanted to budget for language services, but I didn’t know how.	58 (17.1)	154 (45.4)	122 (36.0)	5 (1.5)

### Recommendations to Improve Inclusion

Respondents selected recommendations to improve research inclusion along 3 domains: (1) interpretation and translation services; (2) funding and budgeting; and (3) training and institutional support. Among the total 339 respondents, regarding interpretation and translation, 269 (79.4%) strongly agreed or agreed about the need to increase access to interpretation services, 249 (73.5%) desired training on how to procure interpretation and translation resources for their studies, and 244 (71.9%) reported needing validated measures in non-English languages. The majority of respondents (272 [80.2%]) requested free or low-cost interpretation, free or low-cost translation (265 [78.2%]), and guidance on budgeting (235 [69.3%]).

Furthermore, the total respondents frequently listed greater institutional support and networking as crucial to fostering inclusivity in their research; 249 respondents (73.5%) recommended training around interpretation and translation services, and 229 (67.6%) cited a desire to connect with other researchers who had worked with populations using LOE. A full list of proposed solutions and researcher interests is presented in Table 4. Responses to the open-ended questions included recommendations for improved access to language services, mentorship, awareness of resources, training, and institutional advocacy. Table 5 includes sample responses.

**Table 4.** Proposed Solutions for Greater Inclusion With Researcher Interest

Proposed solution	Researcher respondents supporting proposed solutions, No. (%) (N = 339)
**Interpretation and translation services**	
Access to interpretation (verbal or signed) services	269 (79.4)
Access to translation (written) services	262 (77.3)
Validating measures that are translated into different languages	244 (71.9)
Transcription services that assist in multiple languages	213 (62.8)
Language proficiency testing for bilingual staff	137 (40.4)
I do not need access to additional services	19 (5.6)
**Funding and budgeting**	
Free or low-cost interpretation (spoken or signed)	272 (80.2)
Free or low-cost translation (written)	265 (78.2)
Guidance on budgeting for interpretation and translation services	235 (69.3)
I do not need access to additional financial supports	23 (6.8)
**Training and institutional support**	
Training on how to find interpreter and translation services	249 (73.5)
IRB requirements for participants with limited English proficiency	253 (74.6)
Networking with other researchers who include populations with limited English proficiency in research	229 (67.6)
Knowledge about the languages spoken by residents of the greater Pittsburgh area	167 (49.3)
Help hiring bilingual staff	135 (39.8)
I do not need extra training	6 (1.8)

Abbreviation: IRB, institutional review board.

**Table 5.** Representative Quotations^a^

Common themes for suggested solutions to improve inclusion of participants who use LOE in research	Representative quotations from researcher respondents
Improved language and cultural accessibility (183 unique codes)	“I feel that on-site interpreters would be needed for better communication instead of phone translation services. For consenting of research projects, translational services in-house would be better than via phone.”
	“Staff who speak the language so that it is not just the language but also that culture that we take into consideration for our recruitment, intervention, and data collection procedures.”
	“Speaking with community leaders and understanding the culture [would help improve inclusion].”
Validated measures, recruitment, and consenting (83 unique codes)	“I need patient-reported outcome and neuropsychological test measures validated in other languages; these don’t exist in most cases.”
	“Education on how to recruit, consent, and navigate communication throughout the study.”
Institutional advocacy for inclusion of participants using LOE (76 unique codes)	“I feel like it should be a default—the IRB should ask explicitly how the research team will include non-English speakers. And researchers should have a really good reason as to why they exclude anyone who doesn’t speak English.”
	“I think that more than connections, we need people in positions of ‘power’/decision-makers to get vested in including people of different backgrounds in order to make our research trials more diverse and inclusive.”
Awareness and utilization of available resources (50 unique codes)	“I never realized the resources [my institution] has for participants with limited English proficiency. ‘Marketing,’ if you will, of these services would be helpful!”
	“Education on budgeting and including inclusive language services in grant proposals would also be helpful.”
	“[Information] on why it is important to include these populations and what they can add to research.”
Collaboration and mentorship (48 unique codes)	“Having a group of other researchers…that [has] included non-English speakers into their studies would be very helpful.”
	“A more streamlined way to connect with community leaders of those in the target population. Our study finds it difficult to recruit from those who have English as a second language.”

Abbreviations: IRB, institutional review board; LOE, languages other than English.

^a^ Prompt for open-ended response: “Please describe any other tools or connections that you feel would be helpful for you to improve the inclusion of participants with limited English proficiency in your lab or your research projects.”

### Community Partnership

Fifteen of the 339 respondents (4.4%) reported having partnered with a community organization at least once. These respondents reported 29 unique experiences working with a community partner, 23 (79.3%) of which they rated as either very helpful or helpful. When asked about potential solutions to improve research inclusion, 172 respondents (50.7%) reported a desire to build partnerships with community organizations.

## Discussion

This survey study is, to our knowledge, among the first to describe health researchers’ views on barriers to inclusion of participants who use LOE in research and to identify concrete strategies to disrupt this inequity. We found that around half (50.1%) of researchers reported working with participants using LOE in the past 5 years, which is higher than described in prior literature. Research across various pediatric and adult medical specialties has demonstrated a lack of explicit inclusion of participants using LOE in their studies (eg, pediatric studies for more than 10 years suggest only 9% included participants using LOE),^12^ as well as an increase in explicit exclusion due to lack of English proficiency.^12,13,14,15,16,17,27^ Our findings may show higher rates because those who responded to our survey may be more interested in this topic and thus more likely to have included participants using LOE in their research. Alternatively, it is possible that the difference is related to language inclusion being incompletely reported in most research articles, even when participants who use LOE take part. While further work is needed to better understand these findings, our study suggests that a barrier to inclusion of participants who use LOE may be omitting participant language as a key inclusion or exclusion criterion when conceptualizing a research study.

Our current findings further expand on this literature by describing how inclusion of participants using LOE may occur proactively (eg, from the start) or reactively (eg, when participants using LOE were included after the study began). Among respondents, 41.5% reported including only participants who use LOE after the study began to accommodate interest, rather than proactively as part of study protocols. This further supports the notion that language may not be adequately prioritized when designing research studies and securing funding. Inclusion of participants who use LOE requires time, training, and resources and cannot solely be the responsibility of the researcher.^28^ Investment in multilingual research from institutions and funding sources is needed to ensure that investigators have the proper resources to prioritize a proactively inclusive study design and recruitment.

Budgeting for language services was also reported as a barrier, with the vast majority of respondents recommending financial support to make inclusion of participants using LOE more feasible. While research on budgeting for participants using LOE is limited, some studies have cited high costs of translation and obtaining consent as significant barriers to including participants using LOE.^29,30^ Advanced planning can be helpful in mitigating the cost of language services; however, costs can still be onerous, especially for researchers early in their careers who may not have funding. Better training and clear guidelines may help individuals who want to lead language-inclusive studies, but effectively addressing this barrier will require significant structural change.^28^ Sustainable investment is needed from research institutions and funders to make interpretation and translation services affordable while also ensuring pay equity for those providing language services.

Accessing and using interpretation and translation were commonly cited as barriers to including participants using LOE in research, particularly related to time and scheduling. Additionally, the majority of respondents reported that they had never received training to work with participants who use LOE. Similar findings have been shown in health care, in which clinicians across various roles and specialties report a lack of training to use interpreters and adequately care for patients who use LOE.^31,32^ This lack of training and underuse of interpreters can sometimes lead clinicians to use their own nonproficient language skills or to rely on family members or untrained interpreters to communicate with patients.^33,34,35^ Our findings expand this literature into the realm of research, as researchers similarly report insufficient training and low confidence in their ability to work with participants using LOE. Medical schools and residency programs have developed educational strategies related to equitable language services, including training on partnering with medical interpreters and the professionalization of bilingualism among clinicians through training and proficiency assessment.^36,37^ Research institutions should similarly prioritize training on multilingual research methods, as lack of training is a relatively easy gap to address.

Our results also demonstrated that researchers rarely reported partnering with community-based organizations; however, those who did almost universally found community partnership to be beneficial. Community-partnered research aims to radically rethink how research is conceptualized, conducted, and shared by amplifying community expertise, focusing on capacity sharing, and centering community voice and experiences.^38^ Many benefits of community partnership in research are described in the literature, including more effective recruitment,^39^ improved rigor of the work produced,^40^ and increased trustworthiness to combat the long history of harmful research practices that have exploited marginalized communities.^22,41^ Our results highlight the importance of actively investing in community-partnered work as a key strategy to increase equitable inclusion of individuals using LOE in research.

### Implications

This study sets the stage for future research, practice, and policy. This work should be replicated across multiple sites to better reflect researcher attitudes from a variety of geographic and cultural communities. While this study explored the perspectives of individual researchers, future studies should examine the perspectives of research institutions more broadly, since IRBs and administrations set policies that influence inclusion or exclusion of individuals using LOE in research. Complementary qualitative research is also needed to explore in more detail the barriers elucidated in this study and to create innovative solutions to address those barriers.

This study’s findings have significant policy implications on research practices. Our findings underscore the need for improved training and mentorship for researchers on how to work with participants who use LOE. Developing and disseminating a set of best practice guidelines for working with participants using LOE, cocreated with input from multilingual communities, would allow the rapid spread of knowledge and support to a broad set of researchers. These guidelines should be aligned with a language justice framework,^28^ in which language services are seamlessly integrated into all phases of a research project, power is shared, and language is seen as a strength rather than a barrier.

On an institutional level, it is vital to invest in language-affirming services, through free and low-cost translation, training medical interpreters to support research studies, and promoting recruitment and retention of bilingual research team members. Future work should evaluate the cost-effectiveness of different language services to examine which approaches best balance costs and quality and to assess innovative financial solutions. Institutions should offer greater support, such as providing funding to researchers who proactively include participants who use LOE. Language justice consult services offered by institutions might also be helpful in supporting researchers in multilingual methods. Institutions should also invest in community-partnered research and make engagement in multilingual, community-partnered work prioritized in the promotion and tenure process.

### Limitations

There are several limitations to this exploratory study. We used convenience sampling, creating potential selection bias. In particular, people who elected to complete the survey were likely more interested in the topic of multilingual research compared with potentially eligible respondents more broadly. We also recognize that use of convenience sampling may also limit the ability for every potentially eligible respondent to have the opportunity to complete the survey (eg, some may have not seen the recruitment advertisements). Furthermore, our respondent sample disproportionately identified as White, nonimmigrant, and English-speaking. While such demographics reflect the overall research faculty at the University of Pittsburgh, future similar studies should amplify more diverse perspectives. We also were unable to ask respondents more about the topical focus for their research; although we asked about their school affiliation, there was a significant amount of missing data for that question. This study focused on health-related research within an academic institution; future work should consider the perspectives of researchers conducting other types of work (eg, economics, humanities) and those working outside of academia.

Our team also created the survey rather than using validated measures, as, to our knowledge, no validated surveys that assess researchers’ perspectives about inclusion of participants using LOE are currently available. Barriers to including participants using LOE were selected by the research team and through pilot testing; while our team has extensive experience supporting researchers in conducting multilingual research, we did not have an open-ended question to elicit ideas for additional barriers beyond what were presented. Additionally, we included both PIs and research coordinators. While this may have improved the richness of perspectives, in some cases, PIs and coordinators (or multiple PIs) may have worked on the same project. Our open-ended questions were asked at the end of the survey, and qualitative responses may be influenced by prior quantitative questions.

Finally, this study occurred at a single site, limiting generalizability. However, the region (Pittsburgh, Pennsylvania) where this study took place has seen a rapid increase in immigrant communities over the past decade with only emerging bilingual services compared with established immigrant communities.^42^ Thus, this study provided an opportunity to hear perspectives of researchers who may be less familiar with multilingual methods but who live in a community with a growing population of people who use LOE.

## Conclusions

In this survey study of PIs and research coordinators, multiple barriers to inclusion were found, particularly around scheduling, budgeting, and training. The study also found that institutional support is essential to ensure language inclusivity. Multilevel solutions rooted in a language justice approach are needed to ensure that researchers have the support and resources to increase the linguistic inclusivity of their work, which is an important step toward disrupting health disparities experienced by individuals who use LOE.

## Notes

Supplement 1. **eAppendix 1.** Abridged Survey in Order of Data Presented in Manuscript **eAppendix 2.** Survey in Order Presented to Respondents

Supplement 2.
Data Sharing Statement

## References

[1] Selected social characteristics in the United States. US Census Bureau. 2022. Accessed September 29, 2024. https://data.census.gov/table/ACSDP5Y2022.DP02

[2] Khan A, Yin HS, Brach C, ; Patient and Family Centered I-PASS Health Literacy Subcommittee. Association between parent comfort with English and adverse events among hospitalized children. JAMA Pediatr. 2020;174(12):e203215. doi:10.1001/jamapediatrics.2020.3215 33074313 PMC7573792

[3] Yin HS, Mendelsohn AL, Wolf MS, . Parents’ medication administration errors: role of dosing instruments and health literacy. Arch Pediatr Adolesc Med. 2010;164(2):181-186. doi:10.1001/archpediatrics.2009.269 20124148

[4] Squires A, Ma C, Miner S, Feldman P, Jacobs EA, Jones SA. Assessing the influence of patient language preference on 30 day hospital readmission risk from home health care: a retrospective analysis. Int J Nurs Stud. 2022;125:104093. doi:10.1016/j.ijnurstu.2021.104093 34710627 PMC9282680

[5] Tang EW, Go J, Kwok A, . The relationship between language proficiency and surgical length of stay following cardiac bypass surgery. Eur J Cardiovasc Nurs. 2016;15(6):438-446. doi:10.1177/1474515115596645 26198643

[6] Anand KJ, Sepanski RJ, Giles K, Shah SH, Juarez PD. Pediatric intensive care unit mortality among Latino children before and after a multilevel health care delivery intervention. JAMA Pediatr. 2015;169(4):383-390. doi:10.1001/jamapediatrics.2014.3789 25706478

[7] Schweiberger K, Migliori O, Mbangah M, . “How fluent do I need to be to say I’m fluent?” research experiences of communities that speak languages other than English. Community Health Equity Res Policy. 2025;45(2):111-125. doi:10.1177/2752535X241238095 38486412 PMC13220156

[8] Glickman SW, Ndubuizu A, Weinfurt KP, . Perspective: the case for research justice: inclusion of patients with limited English proficiency in clinical research. Acad Med. 2011;86(3):389-393. doi:10.1097/ACM.0b013e318208289a 21248607

[9] Shea L, Pesa J, Geonnotti G, Powell V, Kahn C, Peters W. Improving diversity in study participation: patient perspectives on barriers, racial differences and the role of communities. Health Expect. 2022;25(4):1979-1987. doi:10.1111/hex.13554 35765232 PMC9327876

[10] Kitchin R. The researched opinions on research: disabled people and disability research. Disabil Soc. 2000;15(1):25-47. doi:10.1080/09687590025757

[11] Schmitz RM, Tyler KA, Robinson BA. “To get the word out”: LGBTQ+ young adults’ motivations for participating in qualitative research studies. J Gay Lesbian Soc Serv. 2019;31(2):242-265. doi:10.1080/10538720.2019.1567430

[12] Chen A, Demaestri S, Schweiberger K, . Inclusion of non–English-speaking participants in pediatric health research: a review. JAMA Pediatr. 2023;177(1):81-88. doi:10.1001/jamapediatrics.2022.3828 36315130 PMC10854994

[13] Anwar A, Dawson-Hahn E, Lion KC, Jimenez ME, Yun K. Exclusion of families who speak languages other than English from federally funded pediatric trials. J Pediatr. 2023;262:113597. doi:10.1016/j.jpeds.2023.113597 37399920 PMC10757988

[14] Zeidan AJ, Smith M, Leff R, . Limited English proficiency as a barrier to inclusion in emergency medicine-based clinical stroke research. J Immigr Minor Health. 2023;25(1):181-189. doi:10.1007/s10903-022-01368-y 35652977

[15] Murray S, Buller AM. Exclusion on grounds of language ability—a reporting gap in health services research? J Health Serv Res Policy. 2007;12(4):205-208. doi:10.1258/135581907782101642 17925071

[16] Brodeur M, Herrick J, Guardioloa J, Richman P. Exclusion of non-English speakers in published emergency medicine research—a comparison of 2004 and 2014. Acta Inform Med. 2017;25(2):112-115. doi:10.5455/aim.2017.25.112-115 28883676 PMC5544464

[17] Chen Q, Sánchez Medina CM, Maher CG, . Almost one in five physiotherapy trials excluded people due to lack of language proficiency: a meta-epidemiological study. J Clin Epidemiol. 2022;152:13-22. doi:10.1016/j.jclinepi.2022.09.007 36150549

[18] George S, Duran N, Norris K. A systematic review of barriers and facilitators to minority research participation among African Americans, Latinos, Asian Americans, and Pacific Islanders. Am J Public Health. 2014;104(2):e16-e31. doi:10.2105/AJPH.2013.301706 24328648 PMC3935672

[19] Owen-Smith AA, Woodyatt C, Sineath RC, . Perceptions of barriers to and facilitators of participation in health research among transgender people. Transgend Health. 2016;1(1):187-196. doi:10.1089/trgh.2016.0023 28861532 PMC5549538

[20] Anderson ML, Riker T, Wilkins AM. Application of the truth and reconciliation model to meaningfully engage deaf sign language users in the research process. Cultur Divers Ethnic Minor Psychol. 2023;29(1):15-23. doi:10.1037/cdp0000445 34197145 PMC8720115

[21] Jindal M, Hua MJ, Hartstein M, Martin M. Trustworthiness, not trust: how systemic racism impacts COVID-19 vaccine receipt. Health Equity. 2023;7(1):380-383. doi:10.1089/heq.2022.0145 37476706 PMC10354722

[22] Henderson C, Scott T, Schinder B, . Shifting the paradigm from participant mistrust to researcher & institutional trustworthiness: a qualitative study of researchers’ perspectives on building trustworthiness with Black communities. Community Health Equity Res Policy. 2024;44(2):127-136. doi:10.1177/0272684X221117710 36125424

[23] Sharma A, Minh Duc NT, Luu Lam Thang T, . A consensus-based Checklist for Reporting of Survey Studies (CROSS). J Gen Intern Med. 2021;36(10):3179-3187. doi:10.1007/s11606-021-06737-1 33886027 PMC8481359

[24] Mora V, Colantuono S, Fanali C, ; SC&RN group. Clinical research coordinators: key components of an efficient clinical trial unit. Contemp Clin Trials Commun. 2023;32:101057. doi:10.1016/j.conctc.2023.101057 36747989 PMC9898615

[25] Yeboah D, McDaniel C, Lion KC. Language matters: why we should reconsider the term “limited English proficiency”. Hosp Pediatr. 2023;13(1):e11-e13. doi:10.1542/hpeds.2022-007014 36464981

[26] Patton MQ. Qualitative Research & Evaluation Methods. 4th ed. Sage; 2015.

[27] Egleston BL, Pedraza O, Wong YN, . Characteristics of clinical trials that require participants to be fluent in English. Clin Trials. 2015;12(6):618-626. doi:10.1177/1740774515592881 26152834 PMC4643363

[28] Doan T, López-Zerón G, Prado G, Ragavan MI. Applying a language justice framework in research: a step toward achieving health equity. Health Promot Pract. 2024;15248399241236182:15248399241236182. doi:10.1177/15248399241236182 38462918

[29] Velez MA, Glenn BA, Garcia-Jimenez M, . Consent document translation expense hinders inclusive clinical trial enrolment. Nature. 2023;620(7975):855-862. doi:10.1038/s41586-023-06382-0 37532930 PMC11046417

[30] Glickman SW, Anstrom KJ, Lin L, . Challenges in enrollment of minority, pediatric, and geriatric patients in emergency and acute care clinical research. Ann Emerg Med. 2008;51(6):775-780.e3. doi:10.1016/j.annemergmed.2007.11.002 18191297

[31] Rajbhandari P, Glick AF, Brown MF, VanGeest J. Communication training for pediatric hospitalists and its impact on clinical practice with families using languages other than English. Acad Pediatr. 2024;24(7):1086-1091. doi:10.1016/j.acap.2023.12.003 38110055

[32] Taira BR, Torres J, Nguyen A, Guo R, Samra S. Language assistance for the care of limited English proficiency (LEP) patients in the emergency department: a survey of providers and staff. J Immigr Minor Health. 2020;22(3):439-447. doi:10.1007/s10903-019-00964-9 31898078

[33] Cowden JD, Thompson DA, Ellzey J, Artman M. Getting past getting by: training culturally and linguistically competent bilingual physicians. J Pediatr. 2012;160(6):891-892.e1. doi:10.1016/j.jpeds.2012.02.032 22624935

[34] Hsieh E. Not just “getting by”: factors influencing providers’ choice of interpreters. J Gen Intern Med. 2015;30(1):75-82. doi:10.1007/s11606-014-3066-8 25338731 PMC4284281

[35] Lion KC, Gritton J, Scannell J, . Patterns and predictors of professional interpreter use in the pediatric emergency department. Pediatrics. 2021;147(2):e20193312. doi:10.1542/peds.2019-3312 33468598 PMC7906072

[36] Vargas Pelaez AF, Ramirez SI, Valdes Sanchez C, . Implementing a medical student interpreter training program as a strategy to developing humanism. BMC Med Educ. 2018;18(1):141. doi:10.1186/s12909-018-1254-7 29914460 PMC6006684

[37] Cowden JD, Martinez FJ, Dickmeyer JJ, Bratcher D. Culture and language coaching for bilingual residents: the first 10 years of the CHiCoS model. Teach Learn Med. 2023;35(5):589-600. doi:10.1080/10401334.2022.2092113 35770421

[38] Fleming PJ, Stone LC, Creary MS, . Antiracism and community-based participatory research: synergies, challenges, and opportunities. Am J Public Health. 2023;113(1):70-78. doi:10.2105/AJPH.2022.307114 36516389 PMC9755941

[39] Horowitz CR, Brenner BL, Lachapelle S, Amara DA, Arniella G. Effective recruitment of minority populations through community-led strategies. Am J Prev Med. 2009;37(6)(suppl 1):S195-S200. doi:10.1016/j.amepre.2009.08.006 19896019 PMC2810631

[40] Balazs CL, Morello-Frosch R. The three R’s: how community based participatory research strengthens the rigor, relevance and reach of science. Environ Justice. 2013;6(1). doi:10.1089/env.2012.0017 24260590 PMC3832061

[41] Krakora M, Townsend T, Castillo Smyntek XA, . From vaccines to vitality: the progression of a community-academic collaboration. Health Promot Pract. 2024;25(1):13-16. doi:10.1177/15248399221137271 36482669

[42] New Americans in Pittsburgh. American Immigration Council. September 11, 2023. Accessed September 30, 2024. https://www.americanimmigrationcouncil.org/research/new-americans-pittsburgh

