# Supplementary material for: Researcher Perceptions of Inclusion of Study Participants Who Use Languages Other Than English
Source: JAMA Netw Open. 2025 Mar 28;8(3):e252380. doi: 10.1001/jamanetworkopen.2025.2380 (PMC11953757; doi:10.1001/jamanetworkopen.2025.2380)
Supplement: Supplement 1. — eAppendix 1. Abridged Survey in Order of Data Presented in Manuscript eAppendix 2. Survey in Order Presented to Respondents [file jamanetwopen-e252380-s001.pdf]

Supplemental Online Content

Hoffman H, Doan TT, Migliori O, et al. Researcher perceptions of inclusion of study participants who use languages other than English. *JAMA Netw Open*. 2025;8(3):e252380. doi:10.1001/jamanetworkopen.2025.2380

eAppendix 1. Abridged Survey in Order of Data Presented in Manuscript
eAppendix 2. Survey in Order Presented to Respondents

This supplemental material has been provided by the authors to give readers additional information about their work.

© 2025 Hoffman H et al. *JAMA Network Open*.

eAppendix 1. Abridged Survey in Order of Data Presented in Manuscript

Are you a researcher at the University of Pittsburgh who has been a principal investigator (PI) on the IRB of at least one human subjects research study in the past 5 years (with or without a faculty mentor) OR have you been a project coordinator on at least one human subjects research study in the past 5 years?
If you have been both a PI and a research coordinator, please take the survey while considering your role as a PI. If you are an undergraduate student, graduate student, post-doctoral student, or staff member, you are eligible to participate as long as you have been a primary investigator or project coordinator on a research study. Please contact Olivia Migliori at miglioriog@upmc.edu if you have any questions.

- ○ Yes, I am taking the survey as a principal investigator (1)
- ○ Yes, I am taking the survey as a project coordinator (2)
- ○ No, I do not fit this description (3)

© 2025 Hoffman H et al. *JAMA Network Open*.

Please share your racial and ethnic background. Please select ALL that apply. Under each category is text space for you to describe more detail, if you would like (e.g., under Asian or Pacific Islander, you could write Nepali).

- ☐ Asian (1) ____
- ☐ Black, African, or African American (2) ____
- ☐ Hispanic, Latino, Latina, Latine or Latinx (3) ____
- ☐ Indigenous, American Indian, or Alaskan Native (4) ____
- ☐ Middle Eastern/North African (5) ____
- ☐ Pacific Islander or Native Hawaiian (6) ____
- ☐ White or Caucasian (7) ____
- ☐ Some other race or ethnicity (please describe) (8) ____
- ☐ Prefer not to say (9)

Please share your gender identity. (Select all that apply.)

- ☐ Cisgender female/ woman (1)
- ☐ Cisgender male/ man (2)
- ☐ Genderqueer (3)

© 2025 Hoffman H et al. *JAMA Network Open*.

☐ Non-binary (4)

☐ Transgender female/ trans woman (5)

☐ Transgender male/ trans man (6)

☐ Prefer to self describe (7) ____

☐ Prefer not to say (8)

Page Break

How long have you been doing human subjects research? Please consider the entire amount of time you have been doing human subjects research as a PI, research assistant, or project coordinator either at the University of Pittsburgh or at another institutions.

○ Less than 1 year (1)

○ 1-3 years (2)

○ 4-6 years (3)

○ 7-10 years (4)

○ 11-15 years (5)

○ 16-20 years (6)

○ 20 or more years (7)

© 2025 Hoffman H et al. *JAMA Network Open*.

Please share what type of research you primarily do. Select all that apply.

- ☐ Basic science (1)
- ☐ Clinical (2)
- ☐ Population health (3)
- ☐ Community-partnered (4)
- ☐ Health services (5)
- ☐ Non-health related human subjects research (please describe) (6) ____
- ☐ Other (please describe) (7) ____

© 2025 Hoffman H et al. *JAMA Network Open*.

Please select all the languages you speak and/or understand at any level, including English.

- ☐ Acholi (1)
- ☐ Arabic (3)
- ☐ American Sign Language (ASL) (2)
- ☐ Cantonese (4)
- ☐ English (5)
- ☐ French (6)
- ☐ German (7)
- ☐ Hindi (8)
- ☐ Japanese (9)
- ☐ Korean (10)
- ☐ Mandarin (11)
- ☐ Nepali (12)
- ☐ Pennsylvania Dutch (13)
- ☐ Portuguese (14)
- ☐ Russian (15)
- ☐ Spanish (16)
- ☐ Swahili (17)

© 2025 Hoffman H et al. *JAMA Network Open*.

- [ ] Urdu (18)
- [ ] Uzbek (19)
- [ ] Vietnamese (20)
- [ ] Additional language 1 (please list only one language) (21)

____

- [ ] Additional language 2 (please list only one language) (22)

____

- [ ] Additional language 3 (please list only one language) (23)

____

Page Break

© 2025 Hoffman H et al. *JAMA Network Open*.

Display This Question:

If Please select all the languages you speak and/or understand at any level, including English. = Acholi

Please select your level of fluency in Acholi.

◯ **Native/Functionally Native:** I converse easily and accurately in all types of situations. Native speakers, including the highly educated, may think that I am a native speaker, too. (1)

◯ **Advanced:** I speak very accurately, and I understand other speakers very accurately. Native speakers have no problem understanding me, but they probably perceive that I am not a native speaker. (2)

◯ **Good:** I speak well enough to participate in most conversations. Native speakers notice some errors in my speech or my understanding, but my errors rarely cause misunderstanding. I have some difficulty communicating necessary health concepts. (3)

◯ **Fair:** I speak and understand well enough to have extended conversations about current events, work, family, or personal life. Native speakers notice many errors in my speech or my understanding. I have difficulty communicating about health care concepts (4)

◯ **Basic:** I speak the language imperfectly and only to a limited degree and in limited situations. I have difficulty in or understanding extended conversations. I am unable to understand or communicate most healthcare concepts. (5)

Display This Question:

If Please select all the languages you speak and/or understand at any level, including English. = Arabic

© 2025 Hoffman H et al. *JAMA Network Open*.

Please select your level of fluency in Arabic.

◯ **Native/Functionally Native:** I converse easily and accurately in all types of situations. Native speakers, including the highly educated, may think that I am a native speaker, too. (1)

◯ **Advanced:** I speak very accurately, and I understand other speakers very accurately. Native speakers have no problem understanding me, but they probably perceive that I am not a native speaker. (2)

◯ **Good:** I speak well enough to participate in most conversations. Native speakers notice some errors in my speech or my understanding, but my errors rarely cause misunderstanding. I have some difficulty communicating necessary health concepts. (3)

◯ **Fair:** I speak and understand well enough to have extended conversations about current events, work, family, or personal life. Native speakers notice many errors in my speech or my understanding. I have difficulty communicating about health care concepts (4)

◯ **Basic:** I speak the language imperfectly and only to a limited degree and in limited situations. I have difficulty in or understanding extended conversations. I am unable to understand or communicate most healthcare concepts. (5)

Display This Question:

If Please select all the languages you speak and/or understand at any level, including English. = American Sign Language (ASL)

X→

© 2025 Hoffman H et al. *JAMA Network Open*.

Please select your level of fluency in American Sign Language.

○ **Native/Functionally Native:** I converse easily and accurately in all types of situations. Native speakers, including the highly educated, may think that I am a native speaker, too. (1)

○ **Advanced:** I speak very accurately, and I understand other speakers very accurately. Native speakers have no problem understanding me, but they probably perceive that I am not a native speaker. (2)

○ **Good:** I speak well enough to participate in most conversations. Native speakers notice some errors in my speech or my understanding, but my errors rarely cause misunderstanding. I have some difficulty communicating necessary health concepts. (3)

○ **Fair:** I speak and understand well enough to have extended conversations about current events, work, family, or personal life. Native speakers notice many errors in my speech or my understanding. I have difficulty communicating about health care concepts (4)

○ **Basic:** I speak the language imperfectly and only to a limited degree and in limited situations. I have difficulty in or understanding extended conversations. I am unable to understand or communicate most healthcare concepts. (5)

Display This Question:

If Please select all the languages you speak and/or understand at any level, including English. = Cantonese

X→

© 2025 Hoffman H et al. *JAMA Network Open*.

Please select your level of fluency in Cantonese.

- ◯ **Native/Functionally Native:** I converse easily and accurately in all types of situations. Native speakers, including the highly educated, may think that I am a native speaker, too. (1)
- ◯ **Advanced:** I speak very accurately, and I understand other speakers very accurately. Native speakers have no problem understanding me, but they probably perceive that I am not a native speaker. (2)
- ◯ **Good:** I speak well enough to participate in most conversations. Native speakers notice some errors in my speech or my understanding, but my errors rarely cause misunderstanding. I have some difficulty communicating necessary health concepts. (3)
- ◯ **Fair:** I speak and understand well enough to have extended conversations about current events, work, family, or personal life. Native speakers notice many errors in my speech or my understanding. I have difficulty communicating about health care concepts (4)
- ◯ **Basic:** I speak the language imperfectly and only to a limited degree and in limited situations. I have difficulty in or understanding extended conversations. I am unable to understand or communicate most healthcare concepts. (5)

© 2025 Hoffman H et al. *JAMA Network Open*.

Please select your level of fluency in English.

◯ **Native/Functionally Native:** I converse easily and accurately in all types of situations. Native speakers, including the highly educated, may think that I am a native speaker, too. (1)

◯ **Advanced:** I speak very accurately, and I understand other speakers very accurately. Native speakers have no problem understanding me, but they probably perceive that I am not a native speaker. (2)

◯ **Good:** I speak well enough to participate in most conversations. Native speakers notice some errors in my speech or my understanding, but my errors rarely cause misunderstanding. I have some difficulty communicating necessary health concepts. (3)

◯ **Fair:** I speak and understand well enough to have extended conversations about current events, work, family, or personal life. Native speakers notice many errors in my speech or my understanding. I have difficulty communicating about health care concepts (4)

◯ **Basic:** I speak the language imperfectly and only to a limited degree and in limited situations. I have difficulty in or understanding extended conversations. I am unable to understand or communicate most healthcare concepts. (5)

Display This Question:

If Please select all the languages you speak and/or understand at any level, including English. = French

X→

© 2025 Hoffman H et al. *JAMA Network Open*.

Please select your level of fluency in French.

- ◯ **Native/Functionally Native:** I converse easily and accurately in all types of situations. Native speakers, including the highly educated, may think that I am a native speaker, too. (1)

- ◯ **Advanced:** I speak very accurately, and I understand other speakers very accurately. Native speakers have no problem understanding me, but they probably perceive that I am not a native speaker. (2)

- ◯ **Good:** I speak well enough to participate in most conversations. Native speakers notice some errors in my speech or my understanding, but my errors rarely cause misunderstanding. I have some difficulty communicating necessary health concepts. (3)

- ◯ **Fair:** I speak and understand well enough to have extended conversations about current events, work, family, or personal life. Native speakers notice many errors in my speech or my understanding. I have difficulty communicating about health care concepts (4)

- ◯ **Basic:** I speak the language imperfectly and only to a limited degree and in limited situations. I have difficulty in or understanding extended conversations. I am unable to understand or communicate most healthcare concepts. (5)

Display This Question:

If Please select all the languages you speak and/or understand at any level, including English. = German

© 2025 Hoffman H et al. *JAMA Network Open*.

Please select your level of fluency in German.

◯ **Native/Functionally Native:** I converse easily and accurately in all types of situations. Native speakers, including the highly educated, may think that I am a native speaker, too. (1)

◯ **Advanced:** I speak very accurately, and I understand other speakers very accurately. Native speakers have no problem understanding me, but they probably perceive that I am not a native speaker. (2)

◯ **Good:** I speak well enough to participate in most conversations. Native speakers notice some errors in my speech or my understanding, but my errors rarely cause misunderstanding. I have some difficulty communicating necessary health concepts. (3)

◯ **Fair:** I speak and understand well enough to have extended conversations about current events, work, family, or personal life. Native speakers notice many errors in my speech or my understanding. I have difficulty communicating about health care concepts (4)

◯ **Basic:** I speak the language imperfectly and only to a limited degree and in limited situations. I have difficulty in or understanding extended conversations. I am unable to understand or communicate most healthcare concepts. (5)

Display This Question:

If Please select all the languages you speak and/or understand at any level, including English. = Hindi

© 2025 Hoffman H et al. *JAMA Network Open*.

Please select your level of fluency in Hindi.

- ◯ **Native/Functionally Native:** I converse easily and accurately in all types of situations. Native speakers, including the highly educated, may think that I am a native speaker, too. (1)
- ◯ **Advanced:** I speak very accurately, and I understand other speakers very accurately. Native speakers have no problem understanding me, but they probably perceive that I am not a native speaker. (2)
- ◯ **Good:** I speak well enough to participate in most conversations. Native speakers notice some errors in my speech or my understanding, but my errors rarely cause misunderstanding. I have some difficulty communicating necessary health concepts. (3)
- ◯ **Fair:** I speak and understand well enough to have extended conversations about current events, work, family, or personal life. Native speakers notice many errors in my speech or my understanding. I have difficulty communicating about health care concepts (4)
- ◯ **Basic:** I speak the language imperfectly and only to a limited degree and in limited situations. I have difficulty in or understanding extended conversations. I am unable to understand or communicate most healthcare concepts. (5)

Display This Question:

If Please select all the languages you speak and/or understand at any level, including English. = Japanese

X→

© 2025 Hoffman H et al. *JAMA Network Open*.

Please select your level of fluency in Japanese.

- **Native/Functionally Native:** I converse easily and accurately in all types of situations. Native speakers, including the highly educated, may think that I am a native speaker, too. (1)
- **Advanced:** I speak very accurately, and I understand other speakers very accurately. Native speakers have no problem understanding me, but they probably perceive that I am not a native speaker. (2)
- **Good:** I speak well enough to participate in most conversations. Native speakers notice some errors in my speech or my understanding, but my errors rarely cause misunderstanding. I have some difficulty communicating necessary health concepts. (3)
- **Fair:** I speak and understand well enough to have extended conversations about current events, work, family, or personal life. Native speakers notice many errors in my speech or my understanding. I have difficulty communicating about health care concepts (4)
- **Basic:** I speak the language imperfectly and only to a limited degree and in limited situations. I have difficulty in or understanding extended conversations. I am unable to understand or communicate most healthcare concepts. (5)

Display This Question:

If Please select all the languages you speak and/or understand at any level, including English. = Korean

© 2025 Hoffman H et al. *JAMA Network Open*.

Please select your level of fluency in Korean.

◯ **Native/Functionally Native:** I converse easily and accurately in all types of situations. Native speakers, including the highly educated, may think that I am a native speaker, too. (1)

◯ **Advanced:** I speak very accurately, and I understand other speakers very accurately. Native speakers have no problem understanding me, but they probably perceive that I am not a native speaker. (2)

◯ **Good:** I speak well enough to participate in most conversations. Native speakers notice some errors in my speech or my understanding, but my errors rarely cause misunderstanding. I have some difficulty communicating necessary health concepts. (3)

◯ **Fair:** I speak and understand well enough to have extended conversations about current events, work, family, or personal life. Native speakers notice many errors in my speech or my understanding. I have difficulty communicating about health care concepts (4)

◯ **Basic:** I speak the language imperfectly and only to a limited degree and in limited situations. I have difficulty in or understanding extended conversations. I am unable to understand or communicate most healthcare concepts. (5)

Display This Question:

If Please select all the languages you speak and/or understand at any level, including English. = Mandarin

X→

© 2025 Hoffman H et al. *JAMA Network Open*.

Please select your level of fluency in Mandarin.

◯ **Native/Functionally Native:** I converse easily and accurately in all types of situations. Native speakers, including the highly educated, may think that I am a native speaker, too. (1)

◯ **Advanced:** I speak very accurately, and I understand other speakers very accurately. Native speakers have no problem understanding me, but they probably perceive that I am not a native speaker. (2)

◯ **Good:** I speak well enough to participate in most conversations. Native speakers notice some errors in my speech or my understanding, but my errors rarely cause misunderstanding. I have some difficulty communicating necessary health concepts. (3)

◯ **Fair:** I speak and understand well enough to have extended conversations about current events, work, family, or personal life. Native speakers notice many errors in my speech or my understanding. I have difficulty communicating about health care concepts (4)

◯ **Basic:** I speak the language imperfectly and only to a limited degree and in limited situations. I have difficulty in or understanding extended conversations. I am unable to understand or communicate most healthcare concepts. (5)

Display This Question:

If Please select all the languages you speak and/or understand at any level, including English. = Nepali

X→

© 2025 Hoffman H et al. *JAMA Network Open*.

Please select your level of fluency in Nepali.

- ◯ **Native/Functionally Native:** I converse easily and accurately in all types of situations. Native speakers, including the highly educated, may think that I am a native speaker, too. (1)
- ◯ **Advanced:** I speak very accurately, and I understand other speakers very accurately. Native speakers have no problem understanding me, but they probably perceive that I am not a native speaker. (2)
- ◯ **Good:** I speak well enough to participate in most conversations. Native speakers notice some errors in my speech or my understanding, but my errors rarely cause misunderstanding. I have some difficulty communicating necessary health concepts. (3)
- ◯ **Fair:** I speak and understand well enough to have extended conversations about current events, work, family, or personal life. Native speakers notice many errors in my speech or my understanding. I have difficulty communicating about health care concepts (4)
- ◯ **Basic:** I speak the language imperfectly and only to a limited degree and in limited situations. I have difficulty in or understanding extended conversations. I am unable to understand or communicate most healthcare concepts. (5)

Display This Question:

If Please select all the languages you speak and/or understand at any level, including English. = Pennsylvania Dutch

© 2025 Hoffman H et al. *JAMA Network Open*.

Please select your level of fluency in Pennsylvania Dutch.

◯ **Native/Functionally Native:** I converse easily and accurately in all types of situations. Native speakers, including the highly educated, may think that I am a native speaker, too. (1)

◯ **Advanced:** I speak very accurately, and I understand other speakers very accurately. Native speakers have no problem understanding me, but they probably perceive that I am not a native speaker. (2)

◯ **Good:** I speak well enough to participate in most conversations. Native speakers notice some errors in my speech or my understanding, but my errors rarely cause misunderstanding. I have some difficulty communicating necessary health concepts. (3)

◯ **Fair:** I speak and understand well enough to have extended conversations about current events, work, family, or personal life. Native speakers notice many errors in my speech or my understanding. I have difficulty communicating about health care concepts (4)

◯ **Basic:** I speak the language imperfectly and only to a limited degree and in limited situations. I have difficulty in or understanding extended conversations. I am unable to understand or communicate most healthcare concepts. (5)

Display This Question:

If Please select all the languages you speak and/or understand at any level, including English. = Portuguese

X→

© 2025 Hoffman H et al. *JAMA Network Open*.

Please select your level of fluency in Portuguese.

◯ **Native/Functionally Native:** I converse easily and accurately in all types of situations. Native speakers, including the highly educated, may think that I am a native speaker, too. (1)

◯ **Advanced:** I speak very accurately, and I understand other speakers very accurately. Native speakers have no problem understanding me, but they probably perceive that I am not a native speaker. (2)

◯ **Good:** I speak well enough to participate in most conversations. Native speakers notice some errors in my speech or my understanding, but my errors rarely cause misunderstanding. I have some difficulty communicating necessary health concepts. (3)

◯ **Fair:** I speak and understand well enough to have extended conversations about current events, work, family, or personal life. Native speakers notice many errors in my speech or my understanding. I have difficulty communicating about health care concepts (4)

◯ **Basic:** I speak the language imperfectly and only to a limited degree and in limited situations. I have difficulty in or understanding extended conversations. I am unable to understand or communicate most healthcare concepts. (5)

Display This Question:

If Please select all the languages you speak and/or understand at any level, including English. = Russian

X→

© 2025 Hoffman H et al. *JAMA Network Open*.

Please select your level of fluency in Russian.

◯ **Native/Functionally Native:** I converse easily and accurately in all types of situations. Native speakers, including the highly educated, may think that I am a native speaker, too. (1)

◯ **Advanced:** I speak very accurately, and I understand other speakers very accurately. Native speakers have no problem understanding me, but they probably perceive that I am not a native speaker. (2)

◯ **Good:** I speak well enough to participate in most conversations. Native speakers notice some errors in my speech or my understanding, but my errors rarely cause misunderstanding. I have some difficulty communicating necessary health concepts. (3)

◯ **Fair:** I speak and understand well enough to have extended conversations about current events, work, family, or personal life. Native speakers notice many errors in my speech or my understanding. I have difficulty communicating about health care concepts (4)

◯ **Basic:** I speak the language imperfectly and only to a limited degree and in limited situations. I have difficulty in or understanding extended conversations. I am unable to understand or communicate most healthcare concepts. (5)

Display This Question:

If Please select all the languages you speak and/or understand at any level, including English. = Spanish

X→

© 2025 Hoffman H et al. *JAMA Network Open*.

Please select your level of fluency in Spanish.

◯ **Native/Functionally Native:** I converse easily and accurately in all types of situations. Native speakers, including the highly educated, may think that I am a native speaker, too. (1)

◯ **Advanced:** I speak very accurately, and I understand other speakers very accurately. Native speakers have no problem understanding me, but they probably perceive that I am not a native speaker. (2)

◯ **Good:** I speak well enough to participate in most conversations. Native speakers notice some errors in my speech or my understanding, but my errors rarely cause misunderstanding. I have some difficulty communicating necessary health concepts. (3)

◯ **Fair:** I speak and understand well enough to have extended conversations about current events, work, family, or personal life. Native speakers notice many errors in my speech or my understanding. I have difficulty communicating about health care concepts (4)

◯ **Basic:** I speak the language imperfectly and only to a limited degree and in limited situations. I have difficulty in or understanding extended conversations. I am unable to understand or communicate most healthcare concepts. (5)

Display This Question:

If Please select all the languages you speak and/or understand at any level, including English. = Swahili

X→

© 2025 Hoffman H et al. *JAMA Network Open*.

Please select your level of fluency in Swahili.

◯ **Native/Functionally Native:** I converse easily and accurately in all types of situations. Native speakers, including the highly educated, may think that I am a native speaker, too. (1)

◯ **Advanced:** I speak very accurately, and I understand other speakers very accurately. Native speakers have no problem understanding me, but they probably perceive that I am not a native speaker. (2)

◯ **Good:** I speak well enough to participate in most conversations. Native speakers notice some errors in my speech or my understanding, but my errors rarely cause misunderstanding. I have some difficulty communicating necessary health concepts. (3)

◯ **Fair:** I speak and understand well enough to have extended conversations about current events, work, family, or personal life. Native speakers notice many errors in my speech or my understanding. I have difficulty communicating about health care concepts (4)

◯ **Basic:** I speak the language imperfectly and only to a limited degree and in limited situations. I have difficulty in or understanding extended conversations. I am unable to understand or communicate most healthcare concepts. (5)

Display This Question:

If Please select all the languages you speak and/or understand at any level, including English. = Urdu

© 2025 Hoffman H et al. *JAMA Network Open*.

Please select your level of fluency in Urdu.

○ **Native/Functionally Native:** I converse easily and accurately in all types of situations. Native speakers, including the highly educated, may think that I am a native speaker, too. (1)

○ **Advanced:** I speak very accurately, and I understand other speakers very accurately. Native speakers have no problem understanding me, but they probably perceive that I am not a native speaker. (2)

○ **Good:** I speak well enough to participate in most conversations. Native speakers notice some errors in my speech or my understanding, but my errors rarely cause misunderstanding. I have some difficulty communicating necessary health concepts. (3)

○ **Fair:** I speak and understand well enough to have extended conversations about current events, work, family, or personal life. Native speakers notice many errors in my speech or my understanding. I have difficulty communicating about health care concepts (4)

○ **Basic:** I speak the language imperfectly and only to a limited degree and in limited situations. I have difficulty in or understanding extended conversations. I am unable to understand or communicate most healthcare concepts. (5)

Display This Question:

If Please select all the languages you speak and/or understand at any level, including English. = Uzbek

X→

© 2025 Hoffman H et al. *JAMA Network Open*.

Please select your level of fluency in Uzbek.

◯ **Native/Functionally Native:** I converse easily and accurately in all types of situations. Native speakers, including the highly educated, may think that I am a native speaker, too. (1)

◯ **Advanced:** I speak very accurately, and I understand other speakers very accurately. Native speakers have no problem understanding me, but they probably perceive that I am not a native speaker. (2)

◯ **Good:** I speak well enough to participate in most conversations. Native speakers notice some errors in my speech or my understanding, but my errors rarely cause misunderstanding. I have some difficulty communicating necessary health concepts. (3)

◯ **Fair:** I speak and understand well enough to have extended conversations about current events, work, family, or personal life. Native speakers notice many errors in my speech or my understanding. I have difficulty communicating about health care concepts (4)

◯ **Basic:** I speak the language imperfectly and only to a limited degree and in limited situations. I have difficulty in or understanding extended conversations. I am unable to understand or communicate most healthcare concepts. (5)

Display This Question:

If Please select all the languages you speak and/or understand at any level, including English. = Vietnamese

© 2025 Hoffman H et al. *JAMA Network Open*.

Please select your level of fluency in Vietnamese

◯ **Native/Functionally Native:** I converse easily and accurately in all types of situations. Native speakers, including the highly educated, may think that I am a native speaker, too. (1)

◯ **Advanced:** I speak very accurately, and I understand other speakers very accurately. Native speakers have no problem understanding me, but they probably perceive that I am not a native speaker. (2)

◯ **Good:** I speak well enough to participate in most conversations. Native speakers notice some errors in my speech or my understanding, but my errors rarely cause misunderstanding. I have some difficulty communicating necessary health concepts. (3)

◯ **Fair:** I speak and understand well enough to have extended conversations about current events, work, family, or personal life. Native speakers notice many errors in my speech or my understanding. I have difficulty communicating about health care concepts (4)

◯ **Basic:** I speak the language imperfectly and only to a limited degree and in limited situations. I have difficulty in or understanding extended conversations. I am unable to understand or communicate most healthcare concepts. (5)

Display This Question:

If Please select all the languages you speak and/or understand at any level, including English. = Additional language 1 (please list only one language)

X→

© 2025 Hoffman H et al. *JAMA Network Open*.

Please select your level of fluency in your additional language (1).

○ **Native/Functionally Native:** I converse easily and accurately in all types of situations. Native speakers, including the highly educated, may think that I am a native speaker, too. (1)

○ **Advanced:** I speak very accurately, and I understand other speakers very accurately. Native speakers have no problem understanding me, but they probably perceive that I am not a native speaker. (2)

○ **Good:** I speak well enough to participate in most conversations. Native speakers notice some errors in my speech or my understanding, but my errors rarely cause misunderstanding. I have some difficulty communicating necessary health concepts. (3)

○ **Fair:** I speak and understand well enough to have extended conversations about current events, work, family, or personal life. Native speakers notice many errors in my speech or my understanding. I have difficulty communicating about health care concepts (4)

○ **Basic:** I speak the language imperfectly and only to a limited degree and in limited situations. I have difficulty in or understanding extended conversations. I am unable to understand or communicate most healthcare concepts. (5)

Display This Question:

If Please select all the languages you speak and/or understand at any level, including English. = Additional language 2 (please list only one language)

X→

© 2025 Hoffman H et al. *JAMA Network Open*.

Please select your level of fluency in your additional language (2).

○ **Native/Functionally Native:** I converse easily and accurately in all types of situations. Native speakers, including the highly educated, may think that I am a native speaker, too. (1)

○ **Advanced:** I speak very accurately, and I understand other speakers very accurately. Native speakers have no problem understanding me, but they probably perceive that I am not a native speaker. (2)

○ **Good:** I speak well enough to participate in most conversations. Native speakers notice some errors in my speech or my understanding, but my errors rarely cause misunderstanding. I have some difficulty communicating necessary health concepts. (3)

○ **Fair:** I speak and understand well enough to have extended conversations about current events, work, family, or personal life. Native speakers notice many errors in my speech or my understanding. I have difficulty communicating about health care concepts (4)

○ **Basic:** I speak the language imperfectly and only to a limited degree and in limited situations. I have difficulty in or understanding extended conversations. I am unable to understand or communicate most healthcare concepts. (5)

Display This Question:

If Please select all the languages you speak and/or understand at any level, including English. = Additional language 3 (please list only one language)

© 2025 Hoffman H et al. *JAMA Network Open*.

Please select your level of fluency in your additional language (3).

- ◯ **Native/Functionally Native:** I converse easily and accurately in all types of situations. Native speakers, including the highly educated, may think that I am a native speaker, too. (1)
- ◯ **Advanced:** I speak very accurately, and I understand other speakers very accurately. Native speakers have no problem understanding me, but they probably perceive that I am not a native speaker. (2)
- ◯ **Good:** I speak well enough to participate in most conversations. Native speakers notice some errors in my speech or my understanding, but my errors rarely cause misunderstanding. I have some difficulty communicating necessary health concepts. (3)
- ◯ **Fair:** I speak and understand well enough to have extended conversations about current events, work, family, or personal life. Native speakers notice many errors in my speech or my understanding. I have difficulty communicating about health care concepts (4)
- ◯ **Basic:** I speak the language imperfectly and only to a limited degree and in limited situations. I have difficulty in or understanding extended conversations. I am unable to understand or communicate most healthcare concepts. (5)

Page Break

Has someone with limited English proficiency participated in one of your human subjects studies <u>in the last 5 years</u>? We consider participation to mean having had any contact with your research team during the processes of recruitment, consenting, or conducting research activities. A participant does not have to have completed all parts of the study in order to be considered a participant for the purposes of this survey.

- ◯ Yes (1)
- ◯ No (2)

End of Block: Researcher info

Start of Block: Experience w/ NES communities in research -YES HAS INCLUDED

© 2025 Hoffman H et al. *JAMA Network Open*.

How many of your human subjects research studies in the past 5 years have included participants with limited English proficiency?

- All (1)
- Most but not all (2)
- About half (3)
- Some but less than half (4)
- None (5)

How did those with limited English proficiency come to participate in your study/studies? Please select all that apply.

- People with limited English proficiency were included in the study inclusion criteria (1)
- People with limited English proficiency were not included in the study inclusion criteria, but one or more were accommodated when they showed interest in participating (2)
- People with limited English proficiency participated in some other way (please describe) (3) ____

© 2025 Hoffman H et al. *JAMA Network Open*.

Training Please indicate how much you agree that the following statements **impact your ability to conduct research with participants with limited English proficiency**. For project coordinators, consider the questions from the perspective of your lab and PI.

Yes, I am taking the survey as a principal investigator

© 2025 Hoffman H et al. *JAMA Network Open*.

	Strongly agree (1)	Somewhat agree (2)	Neutral (3)	Somewhat disagree (4)	Strongly disagree (5)
I don't know what other **languages are commonly spoken** in my area (1)	○	○	○	○	○
My studies are focused on an **English-speaking population**. (3)	○	○	○	○	○
I have **never had someone with limited English proficiency interested** in any of my research studies. (4)	○	○	○	○	○
I anticipate **low retention rates** among participants with limited English proficiency. (18)	○	○	○	○	○
My study team members **only speak English.** (23)	○	○	○	○	○
My study team members speak other languages but **I'm not sure about their level of fluency** in languages other than English. (24)	○	○	○	○	○
I **haven't tried to hire** bilingual/multilingual study team members. (25)	○	○	○	○	○
I have **trouble hiring** bilingual/multilingual staff. (26)	○	○	○	○	○

© 2025 Hoffman H et al. *JAMA Network Open*.

Display This Choice: If Are you a researcher at the University of Pittsburgh who has been a principal investigator (PI) o... = Yes, I am taking the survey as a principal investigator I don't know **where to find a community-based organization** who works with limited English proficient communities. (27)	○	○	○	○	○
Display This Choice: If Are you a researcher at the University of Pittsburgh who has been a principal investigator (PI) o... = Yes, I am taking the survey as a principal investigator I was **not trained** to include people with limited English proficiency into my research. (21)	○	○	○	○	○

© 2025 Hoffman H et al. *JAMA Network Open*.

Display This Choice: If Are you a researcher at the University of Pittsburgh who has been a principal investigator (PI) o... = Yes, I am taking the survey as a principal investigator My **mentors** have not included people with limited English proficiency in their research. (22)	○	○	○	○	○

Interp trans Please indicate how much you agree that the following statements about working with interpreters (spoken/signed) and translators (written) **impact <u>your</u> ability to conduct**

© 2025 Hoffman H et al. *JAMA Network Open*.

research with participants with limited English proficiency. For project coordinators, consider the questions from the perspective of your lab and PI.

	Strongly agree (1)	Somewhat agree (2)	Neutral (3)	Somewhat disagree (4)	Strongly disagree (5)
I don't know **where to start looking** for professional language services. (5)	○	○	○	○	○
**I am not familiar with** using professional interpretation or translation services. (9)	○	○	○	○	○
I **struggle to find** professional language services that offer the language of my target population. (27)	○	○	○	○	○
I anticipate **time/scheduling barriers** when utilizing interpretation/translation services. (24)	○	○	○	○	○
I am **concerned about the quality** of professional interpretation or translation services. (25)	○	○	○	○	○
I believe participants with limited English proficiency are **uncomfortable using interpreters** to communicate with the study team. (23)	○	○	○	○	○

© 2025 Hoffman H et al. *JAMA Network Open*.

Page Break

© 2025 Hoffman H et al. *JAMA Network Open*.

funding/other Please indicate whether or not each of the following statements about funding describe **your experience related to conducting research with participants with limited English proficiency**. For project coordinators, consider the questions from the perspective of your lab and PI.

	True (1)	False (2)	Unknown (3)
I **haven't budgeted** for language services in grant proposals. (11)	○	○	○
I budgeted for language services in grant proposals that were **not funded.** (12)	○	○	○
I wanted to include participants with limited English proficiency but it was **too costly.** (10)	○	○	○
I wanted to budget for language services but I **didn't know how.** (23)	○	○	○

End of Block: Barriers and facilitators to including NES communities in research

Start of Block: Recommendations to improve inclusivity

© 2025 Hoffman H et al. *JAMA Network Open*.

What training do you think you need to improve the inclusion of limited English proficiency participants in your studies? Please select all that apply.

- ☐ Training on how to find interpreter and translation services (1)
- ☐ IRB requirements for participants with limited English proficiency (2)
- ☐ How to provide results back to the community (3)
- ☐ How to build partnerships with community-based organizations (4)
- ☐ Knowledge about the languages spoken by residents of the Greater Pittsburgh Area (5)
- ☐ Validating measures that are translated into different languages (6)
- ☐ Other (please describe) (7) ____
- ☐ I do not need extra training (8)

© 2025 Hoffman H et al. *JAMA Network Open*.

What access to services do you think you need to improve the inclusion of participants with limited English proficiency in your studies? Please select all that apply.

- ☐ Access to interpretation (verbal/signed) services (1)
- ☐ Access to translation (written) services (2)
- ☐ Transcription services that assist in multiple languages (3)
- ☐ Language proficiency testing for bilingual staff (4)
- ☐ Other (please describe) (5) ____
- ☐ I do not need access to additional services (6)

What financial supports do you think you need to improve the inclusion of participants with limited English proficiency in your studies? Please select all that apply.

- ☐ Free or low-cost interpretation (spoken/signed) (1)
- ☐ Free or low-cost translation (written) (2)
- ☐ Guidance on budgeting for interpretation and translation services (3)
- ☐ Other (please describe) (4) ____
- ☐ I do not need access to additional financial supports (5)

© 2025 Hoffman H et al. *JAMA Network Open*.

What networking and support do you think you need to improve the inclusion of participants with limited English proficiency in your studies? Please select all that apply.

- ▢ Help hiring bilingual staff (1)
- ▢ Access to community leaders in the target language community (2)
- ▢ Access to community organizations in the target language community (3)
- ▢ Networking with other researchers who include limited English proficient populations in research (4)
- ▢ Consult service to assist with how best to include participants with limited English proficiency (5)
- ▢ Other (please describe) (6) ____
- ▢ I do not need access to additional networking and supports (7)

Page Break

In 2-3 sentences, please describe any other tools or connections that you feel would be helpful for you to improve the inclusion of participants with limited English proficiency **in your lab or your research projects.**

____

In 2-3 sentences, please describe any other tools or connections that you feel would be helpful to improve the inclusion of participants with limited English proficiency in studies **across the University.**

____

© 2025 Hoffman H et al. *JAMA Network Open*.

Have you ever partnered with a Pittsburgh-based immigrant and refugee serving community organization on a research study in the past 5 years?

- ◯ Yes (1)
- ◯ No (2)

How helpful was the community-based organization?

- ◯ Very helpful (1)
- ◯ Helpful (2)
- ◯ Neither helpful nor unhelpful (3)
- ◯ Unhelpful (4)
- ◯ Very unhelpful (5)

© 2025 Hoffman H et al. *JAMA Network Open*.

eAppendix 2. Survey in Order Presented to Respondents

Q22 **Are you a researcher at the University of Pittsburgh who has been a principal investigator (PI) on the IRB of at least one human subjects research study in the past 5 years (with or without a faculty mentor) OR have you been a project coordinator on at least one human subjects research study in the past 5 years?** If you have been both a PI and a research coordinator, please take the survey while considering your role as a PI. If you are an undergraduate student, graduate student, post-doctoral student, or staff member, you are eligible to participate as long as you have been a primary investigator or project coordinator on a research study. Please contact Olivia Migliori at miglioriog@upmc.edu if you have any questions.

- ○ Yes, I am taking the survey as a principal investigator (1)
- ○ Yes, I am taking the survey as a project coordinator (2)
- ○ No, I do not fit this description (3)

Skip To: End of Block If Are you a researcher at the University of Pittsburgh who has been a principal investigator (PI) o... = No, I do not fit this description

Page Break

© 2025 Hoffman H et al. *JAMA Network Open*.

Q2 **How long have you been doing human subjects research?** Please consider the entire amount of time you have been doing human subjects research as a PI, research assistant, or project coordinator either at the University of Pittsburgh or at another institutions.

- ○ Less than 1 year (1)
- ○ 1-3 years (2)
- ○ 4-6 years (3)
- ○ 7-10 years (4)
- ○ 11-15 years (5)
- ○ 16-20 years (6)
- ○ 20 or more years (7)

Page Break

© 2025 Hoffman H et al. *JAMA Network Open*.

Q53 **Please share your primary role with the University.** If you serve or have served in multiple roles, please select the most recent role in which you have been a PI. If you have not been a PI, please select the label that best corresponds with your role when acting as a project coordinator.

- ○ Professor (1)
- ○ Associate professor (2)
- ○ Assistant professor (3)
- ○ Post doc (4)
- ○ Doctoral candidate (5)
- ○ Medical student (6)
- ○ Masters student (7)
- ○ Undergraduate student (8)
- ○ Resident (9)
- ○ Clinical fellow (10)
- ○ Staff (11)
- ○ Other (please describe) (12) ____

Page Break

© 2025 Hoffman H et al. *JAMA Network Open*.

Q8 Please share what type of research you primarily do. Select all that apply.

- ▢ Basic science (1)
- ▢ Clinical (2)
- ▢ Population health (3)
- ▢ Community-partnered (4)
- ▢ Health services (5)
- ▢ Non-health related human subjects research (please describe) (6) ____
- ▢ Other (please describe) (7) ____

© 2025 Hoffman H et al. *JAMA Network Open*.

Q113 Please select what University of Pittsburgh school you are affiliated with. In the blank, please list your department.

- ○ Arts and Sciences (1) ____
- ○ Dental Medicine (2) ____
- ○ Health and Rehabilitation Sciences (3) ____
- ○ Medicine (4) ____
- ○ Nursing (5) ____
- ○ Public Health (6) ____
- ○ Social Work (7) ____
- ○ Other (Please list school and department) (8) ____

Page Break

© 2025 Hoffman H et al. *JAMA Network Open*.

Q61 Have you conducted research in a city other than Pittsburgh in the past 5 years?

○ Yes (1)

○ No (2)

Display This Question:

If Have you conducted research in a city other than Pittsburgh in the past 5 years? = Yes

Q62 Where did you work other than Pittsburgh in the past 5 years?

____

End of Block: Intro

Start of Block: Researcher info

Q9 Please consider your experiences including participants with limited English proficiency in human subjects research studies for which you were the Principal Investigator or the Project Coordinator in the last 5 years when answering the following questions. **Limited English proficiency**- This can describe participants or communities who do not speak any English at all or who may speak some English but who would request or require language interpretation (verbal/signed) and/or translation (written) services at some or all parts of the research process in order to give full, informed consent and participation.

Page Break

© 2025 Hoffman H et al. *JAMA Network Open*.

Q10 **Has someone with limited English proficiency participated in one of your human subjects studies in the last 5 years?** We consider participation to mean having had any contact with your research team during the processes of recruitment, consenting, or conducting research activities. A participant does not have to have completed all parts of the study in order to be considered a participant for the purposes of this survey.

- ○ Yes (1)
- ○ No (2)

End of Block: Researcher info

Start of Block: Experience w/ NES communities in research -YES HAS INCLUDED

Q54 How did those with limited English proficiency come to participate in your study/studies? Please select all that apply.

- ☐ People with limited English proficiency were included in the study inclusion criteria (1)
- ☐ People with limited English proficiency were not included in the study inclusion criteria, but one or more were accommodated when they showed interest in participating (2)
- ☐ People with limited English proficiency participated in some other way (please describe) (3) ____

Page Break

© 2025 Hoffman H et al. *JAMA Network Open*.

Q36 How many of your human subjects research studies in the past 5 years have included participants with limited English proficiency?

- All (1)
- Most but not all (2)
- About half (3)
- Some but less than half (4)
- None (5)

Page Break

© 2025 Hoffman H et al. *JAMA Network Open*.

Q13 **Individuals who speak which of the following languages have participated in your current or previous studies in the past 5 years?** Please select all that apply. A reminder that for this study,

© 2025 Hoffman H et al. *JAMA Network Open*.

"participation" can refer to any contact with the research team from recruitment, consenting, to conducting research activities.

- ☐ Acholi (1)
- ☐ American Sign Language (2)
- ☐ Arabic (3)
- ☐ Cantonese (4)
- ☐ French (6)
- ☐ German (7)
- ☐ Hindi (8)
- ☐ Japanese (9)
- ☐ Korean (10)
- ☐ Mandarin (11)
- ☐ Nepali (12)
- ☐ Pennsylvania Dutch (13)
- ☐ Portuguese (14)
- ☐ Russian (15)
- ☐ Spanish (16)
- ☐ Swahili (17)
- ☐ Urdu (18)

© 2025 Hoffman H et al. *JAMA Network Open*.

☐ Uzbek (19)

☐ Vietnamese (20)

☐ Other, including other sign languages or indigenous languages (please fill in) (21)
____

Page Break

© 2025 Hoffman H et al. *JAMA Network Open*.

Q12 How do you and your staff determine potential participants' language preferences? Please select all methods that you have used in current or previous research studies within the past 5 years.

- ☐ Don't determine language preference (1)
- ☐ Refer to language preference self-identified in a research repository (2)
- ☐ Refer to language preference documented in the electronic medical record (3)
- ☐ Ask each participant their language preference (4)
- ☐ Ask participants for language preference when there's a perceived communication barrier (5)
- ☐ Ask participant's support person (adult) for the participant's language preference (6)
- ☐ Ask participant's support person (minor child) for the participant's language preference (7)
- ☐ I don't know how my staff does this (8)
- ☐ Other (please describe) (9) ____

Page Break

© 2025 Hoffman H et al. *JAMA Network Open*.

Q51 What types of language preferences do you and your staff ask about? Please select all that apply.

- ☐ Ask participants for their general language preference (1)
- ☐ Ask participants to share all languages that they may speak or understand (2)
- ☐ Ask participants for their language preference for speaking (3)
- ☐ Ask participants for their language preference when writing (4)
- ☐ Ask participants for their language preference when reading (5)
- ☐ Ask participants for their language preference for medical visits (6)
- ☐ I don't know how my staff does this (7)
- ☐ Other (please describe) (8) ____

Page Break

© 2025 Hoffman H et al. *JAMA Network Open*.

Q14 Please consider the language services you and your study team provided during the research process for your studies conducted in the past 5 years. This can include all studies in which people with limited English proficiency participated.

Page Break

© 2025 Hoffman H et al. *JAMA Network Open*.

Q15 **How did your staff communicate with participants with limited English proficiency?** This can refer to any time during the research process from recruitment through completion of research activities. Please consider the methods you used for all of your studies involving participants with limited English proficiency in the past 5 years. Please select all that apply.

- ☐ Communicated with the participants in English (1)
- ☐ Communicated with the participants in their preferred language (2)

Display This Question:

If How did your staff communicate with participants with limited English proficiency? This can refer... = Communicated with the participants in their preferred language

X→

Q111 **What techniques did you and your staff use for verbal/signed language communication with participants in their preferred language?** This can refer to any time during the research process from recruitment through completion of research activities. Please consider the methods

© 2025 Hoffman H et al. *JAMA Network Open*.

you used for all of your studies involving participants with limited English proficiency in the past 5 years. Please select all that apply.

- ☐ No verbal/signed communication provided in their preferred language (1)
- ☐ Professional interpretation services by phone (2)
- ☐ Professional interpretation services in person (3)
- ☐ Professional interpretation services by video (4)
- ☐ Use of online translation service for verbal communication (such as reading Google translate out loud or using Google translate's "text to talk" feature) (5)
- ☐ Interpretation by the participant's minor family member (e.g., child or adolescent) (6)
- ☐ Interpretation by the participant's adult family member or friend (7)
- ☐ Bilingual team member interpretation or communication in target language (8)
- ☐ Bilingual community leaders or community health workers who were part of the study team (9)
- ☐ Other (please describe) (10) ____

Display This Question:

If How did your staff communicate with participants with limited English proficiency? This can refer... = Communicated with the participants in their preferred language

Q40 **What techniques did you and your staff use for written communication with participants in their preferred language?** This can refer to any time during the research process from recruitment through completion of research activities. Please consider the methods you used for

© 2025 Hoffman H et al. *JAMA Network Open*.

all of your studies involving participants with limited English proficiency in the past 5 years. Please select all that apply.

- ☐ No written communication provided in their preferred language (1)
- ☐ Internet translation service (such as Bing or Google translate) (2)
- ☐ Professional translation service (3)
- ☐ Community member translation (4)
- ☐ Bilingual team member translation (5)
- ☐ Asked someone to read the English document aloud in the participant's language (e.g. family member, interpreter, friend, etc.) (6)
- ☐ Other (please describe) (7) ____

Page Break

© 2025 Hoffman H et al. *JAMA Network Open*.

Display This Question:

If What techniques did you and your staff use for verbal/signed language communication with particip... = Bilingual team member interpretation or communication in target language

Or What techniques did you and your staff use for written communication with participants in their p... = Bilingual team member translation

Q74 How did you assess the language ability of the bilingual staff members? Select all that apply.

- ▢ Did not assess (1)
- ▢ Used a fluency test (2)
- ▢ Spoke with them in the target language (3)
- ▢ Staff member had some form of language certification (4)
- ▢ Other (please describe) (5) ____

Page Break

© 2025 Hoffman H et al. *JAMA Network Open*.

Q41 What materials were translated for participants? Please select all that apply.

- ☐ Recruitment flyers (1)
- ☐ Recruitment emails (2)
- ☐ Resource sheet/ FAQ sheet (3)
- ☐ Information scripts/ assent forms (for obtaining verbal consent) (4)
- ☐ Consent forms (for obtaining written consent) (5)
- ☐ Text messages communicating with the study team (6)
- ☐ Emails communicating with the study team (7)
- ☐ Research activity instructions (for activities completed with study team) (8)
- ☐ Research activity instructions (for activities completed at home) (9)
- ☐ Research results (10)
- ☐ Other (please describe) (11) ____

Page Break

© 2025 Hoffman H et al. *JAMA Network Open*.

Q63 Have you ever partnered with a Pittsburgh-based immigrant and refugee serving community organization on a research study in the past 5 years?

- Yes (1)
- No (2)

Page Break

© 2025 Hoffman H et al. *JAMA Network Open*.

Display This Question:

If Have you ever partnered with a Pittsburgh-based immigrant and refugee serving community organizat... = Yes

© 2025 Hoffman H et al. *JAMA Network Open*.

Q46 What Pittsburgh-based organizations have you partnered with on a study in the past 5 years or do you partner with currently? Please select all that apply.

- ☐ All for All (1)
- ☐ ARYSE (2)
- ☐ Asian Pacific Labor Alliance (APALA) Pittsburgh (3)
- ☐ Bhutanese Community Association of Pittsburgh (4)
- ☐ Casa San José (5)
- ☐ Congolese Union of Pittsburgh (6)
- ☐ Family and Immigrant Connections of the Allegheny Intermediate Unit (7)
- ☐ Hello Neighbor (8)
- ☐ Immigrants and Internationals Committee (DHS) (9)
- ☐ Jewish Family and Community Center (JFCS) (10)
- ☐ Latino Community Center (11)
- ☐ Literacy Pittsburgh (12)
- ☐ Organization of Chinese Americans (13)
- ☐ Rangoli Pittsburgh (14)
- ☐ Somali Bantu Community Association of Pittsburgh (15)
- ☐ South Hills Interfaith Movement (SHIM) (16)
- ☐ Other (please describe) (17) ____

© 2025 Hoffman H et al. *JAMA Network Open*.

Page Break

© 2025 Hoffman H et al. *JAMA Network Open*.

Display This Question:

If Has someone with limited English proficiency participated in one of your human subjects studies i... = Yes

Q64 What services in Pittsburgh have you accessed to support the inclusion of people with limited English proficiency in your research in the past 5 years?

- ☐ Community organizations (1)
- ☐ Clinical and Translational Science Institute (CTSI) (2)
- ☐ UPMC Interpreter Services (3)
- ☐ University Center for International Studies (UCIS) (4)
- ☐ Local interpretation/translation service (e.g. Global Wordsmiths, United Language Group) (5)
- ☐ Specific researcher at Pitt who does this work (please name if comfortable) (6) ____
- ☐ Other (please describe) (7) ____
- ☐ The services I used were not Pittsburgh-based (please describe) (8) ____

Page Break

© 2025 Hoffman H et al. *JAMA Network Open*.

Display This Question:

If What services in Pittsburgh have you accessed to support the inclusion of people with limited Eng... = Community organizations

Q65 How helpful was the community-based organization?

- Very helpful (1)
- Helpful (2)
- Neither helpful nor unhelpful (3)
- Unhelpful (4)
- Very unhelpful (5)

Display This Question:

If What services in Pittsburgh have you accessed to support the inclusion of people with limited Eng... = Clinical and Translational Science Institute (CTSI)

Q66 How helpful was the Clinical and Translational Science Institute (CTSI)?

- Very helpful (1)
- Helpful (2)
- Neither helpful nor unhelpful (3)
- Unhelpful (4)
- Very unhelpful (5)

Display This Question:

If What services in Pittsburgh have you accessed to support the inclusion of people with limited Eng... = UPMC Interpreter Services

© 2025 Hoffman H et al. *JAMA Network Open*.

Q67 How helpful were UPMC Interpreter Services?

○ Very helpful (1)

○ Helpful (2)

○ Neither helpful nor unhelpful (3)

○ Unhelpful (4)

○ Very unhelpful (5)

Display This Question:

If What services in Pittsburgh have you accessed to support the inclusion of people with limited Eng... = University Center for International Studies (UCIS)

Q68 How helpful was the University Center for International Studies (UCIS)?

○ Very helpful (1)

○ Helpful (2)

○ Neither helpful nor unhelpful (3)

○ Unhelpful (4)

○ Very unhelpful (5)

Display This Question:

If What services in Pittsburgh have you accessed to support the inclusion of people with limited Eng... = Local interpretation/translation service (e.g. Global Wordsmiths, United Language Group)

© 2025 Hoffman H et al. *JAMA Network Open*.

Q69 How helpful was the local interpretation/translation service (e.g. Global Wordsmiths, United Language Group)?

- ◯ Very helpful (1)
- ◯ Helpful (2)
- ◯ Neither helpful nor unhelpful (3)
- ◯ Unhelpful (4)
- ◯ Very unhelpful (5)

Display This Question:

If What services in Pittsburgh have you accessed to support the inclusion of people with limited Eng... = Specific researcher at Pitt who does this work (please name if comfortable)

Q70 How helpful was the specific researcher at Pitt who does this work?

- ◯ Very helpful (1)
- ◯ Helpful (2)
- ◯ Neither helpful nor unhelpful (3)
- ◯ Unhelpful (4)
- ◯ Very unhelpful (5)

Display This Question:

If What services in Pittsburgh have you accessed to support the inclusion of people with limited Eng... = Other (please describe)

© 2025 Hoffman H et al. *JAMA Network Open*.

Q71 How helpful was the other service you utilized?

- Very helpful (1)
- Helpful (2)
- Neither helpful nor unhelpful (3)
- Unhelpful (4)
- Very unhelpful (5)

Page Break

© 2025 Hoffman H et al. *JAMA Network Open*.

Display This Question:

If Have you conducted research in a city other than Pittsburgh in the past 5 years? = Yes

Q72 You previously stated that you worked and conducted research in a city other than Pittsburgh in the last 5 years. Please describe any community organizations, services, or resources that you may have accessed about incorporating participants with limited English proficiency into your research in that city. Please include how helpful those resources were.

____

End of Block: Experience w/ NES communities in research -YES HAS INCLUDED

Start of Block: Experiences w/ NES - NEVER HAS INCLUDED

Q55 Have you ever tried to recruit people with limited English proficiency without success?

- I have never attempted to do so for any of my studies. (1)
- I have attempted to do so for at least one study without success. (2)

End of Block: Experiences w/ NES - NEVER HAS INCLUDED

Start of Block: Knowledge about best practices for language services

Q11 **How often has one's ability to speak English been listed as an eligibility requirement for participation on all your research studies in the past 5 years?** For example, English fluency or ability is listed as an inclusion criteria or lack of English fluency or ability is listed as an exclusion criteria.

- I always list English ability as an eligibility requirement. (1)
- I usually list English ability as an eligibility requirement. (2)
- I sometimes list English ability as an eligibility requirement and sometimes don't. (3)
- I rarely list English ability as an eligibility requirement. (4)
- I never list English ability as an eligibility requirement. (5)
- I don't include language in my inclusion or exclusion criteria at all. (6)

© 2025 Hoffman H et al. *JAMA Network Open*.

Page Break

© 2025 Hoffman H et al. *JAMA Network Open*.

Q34 How confident do you feel in your knowledge about best practices for the inclusion of participants with limited English proficiency in research?

- ○ Extremely confident (1)
- ○ Somewhat confident (2)
- ○ Neither confident nor unconfident (3)
- ○ Somewhat unconfident (4)
- ○ Extremely unconfident (5)

Page Break

Q52 Have you **ever** utilized interpretation (verbal/signed) or translation (written) services previously? (This can be outside of the context of research, including in your personal life or in prior professional experiences.)

- ○ Yes, I've used both interpretation and translation services (1)
- ○ Yes, I've used interpretation but not translation services (2)
- ○ Yes, I've used translation but not interpretation services (3)
- ○ No, I've never used interpretation or translation services (4)

Page Break

© 2025 Hoffman H et al. *JAMA Network Open*.

Q37 How comfortable would you feel using an interpreter (verbal/signed) for research activities?

- Extremely comfortable (1)
- Somewhat comfortable (2)
- Neither comfortable nor uncomfortable (3)
- Somewhat uncomfortable (4)
- Extremely uncomfortable (5)

Q47 How comfortable would you feel working with a translation (written) company for research activities?

- Extremely comfortable (1)
- Somewhat comfortable (2)
- Neither comfortable nor uncomfortable (3)
- Somewhat uncomfortable (4)
- Extremely uncomfortable (5)

End of Block: Knowledge about best practices for language services

---

Start of Block: Barriers and facilitators to including NES communities in research

© 2025 Hoffman H et al. *JAMA Network Open*.

Q32-Training Please indicate how much you agree that the following statements **impact your ability to conduct research with participants with limited English proficiency**. For project coordinators, consider the questions from the perspective of your lab and PI.

Display This Choice:

If Are you a researcher at the University of Pittsburgh who has been a principal investigator (PI) o... = Yes, I am taking the survey as a principal investigator

Display This Choice:

If Are you a researcher at the University of Pittsburgh who has been a principal investigator (PI) o... = Yes, I am taking the survey as a principal investigator

Display This Choice:

If Are you a researcher at the University of Pittsburgh who has been a principal investigator (PI) o... = Yes, I am taking the survey as a principal investigator

© 2025 Hoffman H et al. *JAMA Network Open*.

	Strongly agree (1)	Somewhat agree (2)	Neutral (3)	Somewhat disagree (4)	Strongly disagree (5)
I don't know what other **languages are commonly spoken** in my area (1)	○	○	○	○	○
My studies are focused on an **English-speaking population**. (3)	○	○	○	○	○
I have **never had someone with limited English proficiency interested** in any of my research studies. (4)	○	○	○	○	○
I anticipate **low retention rates** among participants with limited English proficiency. (18)	○	○	○	○	○
My study team members **only speak English.** (23)	○	○	○	○	○
My study team members speak other languages but **I'm not sure about their level of fluency** in languages other than English. (24)	○	○	○	○	○
I **haven't tried to hire** bilingual/multilingual study team members. (25)	○	○	○	○	○
I have **trouble hiring** bilingual/multilingual staff. (26)	○	○	○	○	○

© 2025 Hoffman H et al. *JAMA Network Open*.

Display This Choice: *If Are you a researcher at the University of Pittsburgh who has been a principal investigator (PI) o... = Yes, I am taking the survey as a principal investigator* I don't know **where to find a community-based organization** who works with limited English proficient communities. (27)	○	○	○	○	○
Display This Choice: *If Are you a researcher at the University of Pittsburgh who has been a principal investigator (PI) o... = Yes, I am taking the survey as a principal investigator* I was **not trained** to include people with limited English proficiency into my research. (21)	○	○	○	○	○
Display This Choice: *If Are you a researcher at the University of Pittsburgh who has been a principal investigator (PI) o... = Yes, I am taking the survey as a principal investigator* My **mentors** have not included people with limited English proficiency in their research. (22)	○	○	○	○	○

© 2025 Hoffman H et al. *JAMA Network Open*.

Page Break

© 2025 Hoffman H et al. *JAMA Network Open*.

Q77-Interp trans Please indicate how much you agree that the following statements about working with interpreters (spoken/signed) and translators (written) **impact your ability to conduct**

© 2025 Hoffman H et al. *JAMA Network Open*.

research with participants with limited English proficiency. For project coordinators, consider the questions from the perspective of your lab and PI.

	Strongly agree (1)	Somewhat agree (2)	Neutral (3)	Somewhat disagree (4)	Strongly disagree (5)
I don't know **where to start looking** for professional language services. (5)	○	○	○	○	○
**I am not familiar with** using professional interpretation or translation services. (9)	○	○	○	○	○
I **struggle to find** professional language services that offer the language of my target population. (27)	○	○	○	○	○
I anticipate **time/scheduling barriers** when utilizing interpretation/translation services. (24)	○	○	○	○	○
I am **concerned about the quality** of professional interpretation or translation services. (25)	○	○	○	○	○
I believe participants with limited English proficiency are **uncomfortable using interpreters** to communicate with the study team. (23)	○	○	○	○	○

© 2025 Hoffman H et al. *JAMA Network Open*.

Page Break

© 2025 Hoffman H et al. *JAMA Network Open*.

Q78- funding/other Please indicate whether or not each of the following statements about funding describe **your experience related to conducting research with participants with limited English proficiency**. For project coordinators, consider the questions from the perspective of your lab and PI.

	True (1)	False (2)	Unknown (3)
I **haven't budgeted** for language services in grant proposals. (11)	○	○	○
I budgeted for language services in grant proposals that were **not funded.** (12)	○	○	○
I wanted to include participants with limited English proficiency but it was **too costly.** (10)	○	○	○
I wanted to budget for language services but **I didn't know how.** (23)	○	○	○

End of Block: Barriers and facilitators to including NES communities in research

---

Start of Block: Recommendations to improve inclusivity

© 2025 Hoffman H et al. *JAMA Network Open*.

Q20 What training do you think you need to improve the inclusion of limited English proficiency participants in your studies? Please select all that apply.

- ☐ Training on how to find interpreter and translation services (1)
- ☐ IRB requirements for participants with limited English proficiency (2)
- ☐ How to provide results back to the community (3)
- ☐ How to build partnerships with community-based organizations (4)
- ☐ Knowledge about the languages spoken by residents of the Greater Pittsburgh Area (5)
- ☐ Validating measures that are translated into different languages (6)
- ☐ Other (please describe) (7) ____
- ☐ I do not need extra training (8)

Q57 What access to services do you think you need to improve the inclusion of participants with limited English proficiency in your studies? Please select all that apply.

- ☐ Access to interpretation (verbal/signed) services (1)
- ☐ Access to translation (written) services (2)
- ☐ Transcription services that assist in multiple languages (3)
- ☐ Language proficiency testing for bilingual staff (4)
- ☐ Other (please describe) (5) ____
- ☐ I do not need access to additional services (6)

© 2025 Hoffman H et al. *JAMA Network Open*.

Q58 What financial supports do you think you need to improve the inclusion of participants with limited English proficiency in your studies? Please select all that apply.

- ☐ Free or low-cost interpretation (spoken/signed) (1)
- ☐ Free or low-cost translation (written) (2)
- ☐ Guidance on budgeting for interpretation and translation services (3)
- ☐ Other (please describe) (4) ____
- ☐ I do not need access to additional financial supports (5)

© 2025 Hoffman H et al. *JAMA Network Open*.

Q59 What networking and support do you think you need to improve the inclusion of participants with limited English proficiency in your studies? Please select all that apply.

- ☐ Help hiring bilingual staff (1)
- ☐ Access to community leaders in the target language community (2)
- ☐ Access to community organizations in the target language community (3)
- ☐ Networking with other researchers who include limited English proficient populations in research (4)
- ☐ Consult service to assist with how best to include participants with limited English proficiency (5)
- ☐ Other (please describe) (6) ____
- ☐ I do not need access to additional networking and supports (7)

Page Break

© 2025 Hoffman H et al. *JAMA Network Open*.

Q26 In 2-3 sentences, please describe any other tools or connections that you feel would be helpful for you to improve the inclusion of participants with limited English proficiency **in your lab or your research projects.**

____

Q38 In 2-3 sentences, please describe any other tools or connections that you feel would be helpful to improve the inclusion of participants with limited English proficiency in studies **across the University.**

____

End of Block: Recommendations to improve inclusivity

Start of Block: Demographics

Q97 Thank you for your feedback. This last section will ask you to provide some demographic information about yourself.

Page Break

© 2025 Hoffman H et al. *JAMA Network Open*.

Q3 Please share your gender identity. (Select all that apply.)

- ☐ Cisgender female/ woman (1)
- ☐ Cisgender male/ man (2)
- ☐ Genderqueer (3)
- ☐ Non-binary (4)
- ☐ Transgender female/ trans woman (5)
- ☐ Transgender male/ trans man (6)
- ☐ Prefer to self describe (7) ____
- ☐ Prefer not to say (8)

Page Break

© 2025 Hoffman H et al. *JAMA Network Open*.

Q4 Please share your racial and ethnic background. Please select ALL that apply. Under each category is text space for you to describe more detail, if you would like (e.g., under Asian or Pacific Islander, you could write Nepali).

- ☐ Asian (1) ____
- ☐ Black, African, or African American (2) ____
- ☐ Hispanic, Latino, Latina, Latine or Latinx (3) ____
- ☐ Indigenous, American Indian, or Alaskan Native (4) ____
- ☐ Middle Eastern/North African (5) ____
- ☐ Pacific Islander or Native Hawaiian (6) ____
- ☐ White or Caucasian (7) ____
- ☐ Some other race or ethnicity (please describe) (8) ____
- ☐ Prefer not to say (9)

© 2025 Hoffman H et al. *JAMA Network Open*.

Q79 Please select all the languages you speak and/or understand at any level, including English.

- ☐ Acholi (1)
- ☐ Arabic (3)
- ☐ American Sign Language (ASL) (2)
- ☐ Cantonese (4)
- ☐ English (5)
- ☐ French (6)
- ☐ German (7)
- ☐ Hindi (8)
- ☐ Japanese (9)
- ☐ Korean (10)
- ☐ Mandarin (11)
- ☐ Nepali (12)
- ☐ Pennsylvania Dutch (13)
- ☐ Portuguese (14)
- ☐ Russian (15)
- ☐ Spanish (16)
- ☐ Swahili (17)

© 2025 Hoffman H et al. *JAMA Network Open*.

- [ ] Urdu (18)
- [ ] Uzbek (19)
- [ ] Vietnamese (20)
- [ ] Additional language 1 (please list only one language) (21) ____
- [ ] Additional language 2 (please list only one language) (22) ____
- [ ] Additional language 3 (please list only one language) (23) ____

Page Break

© 2025 Hoffman H et al. *JAMA Network Open*.

Display This Question:

If Please select all the languages you speak and/or understand at any level, including English. = Acholi

X→

Q98 Please select your level of fluency in Acholi.

◯ **Native/Functionally Native:** I converse easily and accurately in all types of situations. Native speakers, including the highly educated, may think that I am a native speaker, too. (1)

◯ **Advanced:** I speak very accurately, and I understand other speakers very accurately. Native speakers have no problem understanding me, but they probably perceive that I am not a native speaker. (2)

◯ **Good:** I speak well enough to participate in most conversations. Native speakers notice some errors in my speech or my understanding, but my errors rarely cause misunderstanding. I have some difficulty communicating necessary health concepts. (3)

◯ **Fair:** I speak and understand well enough to have extended conversations about current events, work, family, or personal life. Native speakers notice many errors in my speech or my understanding. I have difficulty communicating about health care concepts (4)

◯ **Basic:** I speak the language imperfectly and only to a limited degree and in limited situations. I have difficulty in or understanding extended conversations. I am unable to understand or communicate most healthcare concepts. (5)

Display This Question:

If Please select all the languages you speak and/or understand at any level, including English. = Arabic

X→

© 2025 Hoffman H et al. *JAMA Network Open*.

Q80 Please select your level of fluency in Arabic.

◯ **Native/Functionally Native:** I converse easily and accurately in all types of situations. Native speakers, including the highly educated, may think that I am a native speaker, too. (1)

◯ **Advanced:** I speak very accurately, and I understand other speakers very accurately. Native speakers have no problem understanding me, but they probably perceive that I am not a native speaker. (2)

◯ **Good:** I speak well enough to participate in most conversations. Native speakers notice some errors in my speech or my understanding, but my errors rarely cause misunderstanding. I have some difficulty communicating necessary health concepts. (3)

◯ **Fair:** I speak and understand well enough to have extended conversations about current events, work, family, or personal life. Native speakers notice many errors in my speech or my understanding. I have difficulty communicating about health care concepts (4)

◯ **Basic:** I speak the language imperfectly and only to a limited degree and in limited situations. I have difficulty in or understanding extended conversations. I am unable to understand or communicate most healthcare concepts. (5)

Display This Question:

If Please select all the languages you speak and/or understand at any level, including English. = American Sign Language (ASL)

X→

© 2025 Hoffman H et al. *JAMA Network Open*.

Q96 Please select your level of fluency in American Sign Language.

○ **Native/Functionally Native:** I converse easily and accurately in all types of situations. Native speakers, including the highly educated, may think that I am a native speaker, too. (1)

○ **Advanced:** I speak very accurately, and I understand other speakers very accurately. Native speakers have no problem understanding me, but they probably perceive that I am not a native speaker. (2)

○ **Good:** I speak well enough to participate in most conversations. Native speakers notice some errors in my speech or my understanding, but my errors rarely cause misunderstanding. I have some difficulty communicating necessary health concepts. (3)

○ **Fair:** I speak and understand well enough to have extended conversations about current events, work, family, or personal life. Native speakers notice many errors in my speech or my understanding. I have difficulty communicating about health care concepts (4)

○ **Basic:** I speak the language imperfectly and only to a limited degree and in limited situations. I have difficulty in or understanding extended conversations. I am unable to understand or communicate most healthcare concepts. (5)

Display This Question:

If Please select all the languages you speak and/or understand at any level, including English. = Cantonese

X→

© 2025 Hoffman H et al. *JAMA Network Open*.

Q84 Please select your level of fluency in Cantonese.

- ○ **Native/Functionally Native:** I converse easily and accurately in all types of situations. Native speakers, including the highly educated, may think that I am a native speaker, too. (1)
- ○ **Advanced:** I speak very accurately, and I understand other speakers very accurately. Native speakers have no problem understanding me, but they probably perceive that I am not a native speaker. (2)
- ○ **Good:** I speak well enough to participate in most conversations. Native speakers notice some errors in my speech or my understanding, but my errors rarely cause misunderstanding. I have some difficulty communicating necessary health concepts. (3)
- ○ **Fair:** I speak and understand well enough to have extended conversations about current events, work, family, or personal life. Native speakers notice many errors in my speech or my understanding. I have difficulty communicating about health care concepts (4)
- ○ **Basic:** I speak the language imperfectly and only to a limited degree and in limited situations. I have difficulty in or understanding extended conversations. I am unable to understand or communicate most healthcare concepts. (5)

© 2025 Hoffman H et al. *JAMA Network Open*.

Q85 Please select your level of fluency in English.

- ◯ **Native/Functionally Native:** I converse easily and accurately in all types of situations. Native speakers, including the highly educated, may think that I am a native speaker, too. (1)
- ◯ **Advanced:** I speak very accurately, and I understand other speakers very accurately. Native speakers have no problem understanding me, but they probably perceive that I am not a native speaker. (2)
- ◯ **Good:** I speak well enough to participate in most conversations. Native speakers notice some errors in my speech or my understanding, but my errors rarely cause misunderstanding. I have some difficulty communicating necessary health concepts. (3)
- ◯ **Fair:** I speak and understand well enough to have extended conversations about current events, work, family, or personal life. Native speakers notice many errors in my speech or my understanding. I have difficulty communicating about health care concepts (4)
- ◯ **Basic:** I speak the language imperfectly and only to a limited degree and in limited situations. I have difficulty in or understanding extended conversations. I am unable to understand or communicate most healthcare concepts. (5)

Display This Question:

If Please select all the languages you speak and/or understand at any level, including English. = French

X→

© 2025 Hoffman H et al. *JAMA Network Open*.

Q86 Please select your level of fluency in French.

◯ **Native/Functionally Native:** I converse easily and accurately in all types of situations. Native speakers, including the highly educated, may think that I am a native speaker, too. (1)

◯ **Advanced:** I speak very accurately, and I understand other speakers very accurately. Native speakers have no problem understanding me, but they probably perceive that I am not a native speaker. (2)

◯ **Good:** I speak well enough to participate in most conversations. Native speakers notice some errors in my speech or my understanding, but my errors rarely cause misunderstanding. I have some difficulty communicating necessary health concepts. (3)

◯ **Fair:** I speak and understand well enough to have extended conversations about current events, work, family, or personal life. Native speakers notice many errors in my speech or my understanding. I have difficulty communicating about health care concepts (4)

◯ **Basic:** I speak the language imperfectly and only to a limited degree and in limited situations. I have difficulty in or understanding extended conversations. I am unable to understand or communicate most healthcare concepts. (5)

Display This Question:

If Please select all the languages you speak and/or understand at any level, including English. = German

© 2025 Hoffman H et al. *JAMA Network Open*.

Q87 Please select your level of fluency in German.

◯ **Native/Functionally Native:** I converse easily and accurately in all types of situations. Native speakers, including the highly educated, may think that I am a native speaker, too. (1)

◯ **Advanced:** I speak very accurately, and I understand other speakers very accurately. Native speakers have no problem understanding me, but they probably perceive that I am not a native speaker. (2)

◯ **Good:** I speak well enough to participate in most conversations. Native speakers notice some errors in my speech or my understanding, but my errors rarely cause misunderstanding. I have some difficulty communicating necessary health concepts. (3)

◯ **Fair:** I speak and understand well enough to have extended conversations about current events, work, family, or personal life. Native speakers notice many errors in my speech or my understanding. I have difficulty communicating about health care concepts (4)

◯ **Basic:** I speak the language imperfectly and only to a limited degree and in limited situations. I have difficulty in or understanding extended conversations. I am unable to understand or communicate most healthcare concepts. (5)

Display This Question:

If Please select all the languages you speak and/or understand at any level, including English. = Hindi

X→

© 2025 Hoffman H et al. *JAMA Network Open*.

Q88 Please select your level of fluency in Hindi.

- **Native/Functionally Native:** I converse easily and accurately in all types of situations. Native speakers, including the highly educated, may think that I am a native speaker, too. (1)
- **Advanced:** I speak very accurately, and I understand other speakers very accurately. Native speakers have no problem understanding me, but they probably perceive that I am not a native speaker. (2)
- **Good:** I speak well enough to participate in most conversations. Native speakers notice some errors in my speech or my understanding, but my errors rarely cause misunderstanding. I have some difficulty communicating necessary health concepts. (3)
- **Fair:** I speak and understand well enough to have extended conversations about current events, work, family, or personal life. Native speakers notice many errors in my speech or my understanding. I have difficulty communicating about health care concepts (4)
- **Basic:** I speak the language imperfectly and only to a limited degree and in limited situations. I have difficulty in or understanding extended conversations. I am unable to understand or communicate most healthcare concepts. (5)

Display This Question:

If Please select all the languages you speak and/or understand at any level, including English. = Japanese

X→

© 2025 Hoffman H et al. *JAMA Network Open*.

Q100 Please select your level of fluency in Japanese.

◯ **Native/Functionally Native:** I converse easily and accurately in all types of situations. Native speakers, including the highly educated, may think that I am a native speaker, too. (1)

◯ **Advanced:** I speak very accurately, and I understand other speakers very accurately. Native speakers have no problem understanding me, but they probably perceive that I am not a native speaker. (2)

◯ **Good:** I speak well enough to participate in most conversations. Native speakers notice some errors in my speech or my understanding, but my errors rarely cause misunderstanding. I have some difficulty communicating necessary health concepts. (3)

◯ **Fair:** I speak and understand well enough to have extended conversations about current events, work, family, or personal life. Native speakers notice many errors in my speech or my understanding. I have difficulty communicating about health care concepts (4)

◯ **Basic:** I speak the language imperfectly and only to a limited degree and in limited situations. I have difficulty in or understanding extended conversations. I am unable to understand or communicate most healthcare concepts. (5)

Display This Question:

If Please select all the languages you speak and/or understand at any level, including English. = Korean

X→

© 2025 Hoffman H et al. *JAMA Network Open*.

Q101 Please select your level of fluency in Korean.

○ **Native/Functionally Native:** I converse easily and accurately in all types of situations. Native speakers, including the highly educated, may think that I am a native speaker, too. (1)

○ **Advanced:** I speak very accurately, and I understand other speakers very accurately. Native speakers have no problem understanding me, but they probably perceive that I am not a native speaker. (2)

○ **Good:** I speak well enough to participate in most conversations. Native speakers notice some errors in my speech or my understanding, but my errors rarely cause misunderstanding. I have some difficulty communicating necessary health concepts. (3)

○ **Fair:** I speak and understand well enough to have extended conversations about current events, work, family, or personal life. Native speakers notice many errors in my speech or my understanding. I have difficulty communicating about health care concepts (4)

○ **Basic:** I speak the language imperfectly and only to a limited degree and in limited situations. I have difficulty in or understanding extended conversations. I am unable to understand or communicate most healthcare concepts. (5)

Display This Question:

If Please select all the languages you speak and/or understand at any level, including English. = Mandarin

X→

© 2025 Hoffman H et al. *JAMA Network Open*.

Q102 Please select your level of fluency in Mandarin.

○ **Native/Functionally Native:** I converse easily and accurately in all types of situations. Native speakers, including the highly educated, may think that I am a native speaker, too. (1)

○ **Advanced:** I speak very accurately, and I understand other speakers very accurately. Native speakers have no problem understanding me, but they probably perceive that I am not a native speaker. (2)

○ **Good:** I speak well enough to participate in most conversations. Native speakers notice some errors in my speech or my understanding, but my errors rarely cause misunderstanding. I have some difficulty communicating necessary health concepts. (3)

○ **Fair:** I speak and understand well enough to have extended conversations about current events, work, family, or personal life. Native speakers notice many errors in my speech or my understanding. I have difficulty communicating about health care concepts (4)

○ **Basic:** I speak the language imperfectly and only to a limited degree and in limited situations. I have difficulty in or understanding extended conversations. I am unable to understand or communicate most healthcare concepts. (5)

Display This Question:

If Please select all the languages you speak and/or understand at any level, including English. = Nepali

© 2025 Hoffman H et al. *JAMA Network Open*.

Q89 Please select your level of fluency in Nepali.

◯ **Native/Functionally Native:** I converse easily and accurately in all types of situations. Native speakers, including the highly educated, may think that I am a native speaker, too. (1)

◯ **Advanced:** I speak very accurately, and I understand other speakers very accurately. Native speakers have no problem understanding me, but they probably perceive that I am not a native speaker. (2)

◯ **Good:** I speak well enough to participate in most conversations. Native speakers notice some errors in my speech or my understanding, but my errors rarely cause misunderstanding. I have some difficulty communicating necessary health concepts. (3)

◯ **Fair:** I speak and understand well enough to have extended conversations about current events, work, family, or personal life. Native speakers notice many errors in my speech or my understanding. I have difficulty communicating about health care concepts (4)

◯ **Basic:** I speak the language imperfectly and only to a limited degree and in limited situations. I have difficulty in or understanding extended conversations. I am unable to understand or communicate most healthcare concepts. (5)

Display This Question:

If Please select all the languages you speak and/or understand at any level, including English. = Pennsylvania Dutch

X→

© 2025 Hoffman H et al. *JAMA Network Open*.

Q103 Please select your level of fluency in Pennsylvania Dutch.

○ **Native/Functionally Native:** I converse easily and accurately in all types of situations. Native speakers, including the highly educated, may think that I am a native speaker, too. (1)

○ **Advanced:** I speak very accurately, and I understand other speakers very accurately. Native speakers have no problem understanding me, but they probably perceive that I am not a native speaker. (2)

○ **Good:** I speak well enough to participate in most conversations. Native speakers notice some errors in my speech or my understanding, but my errors rarely cause misunderstanding. I have some difficulty communicating necessary health concepts. (3)

○ **Fair:** I speak and understand well enough to have extended conversations about current events, work, family, or personal life. Native speakers notice many errors in my speech or my understanding. I have difficulty communicating about health care concepts (4)

○ **Basic:** I speak the language imperfectly and only to a limited degree and in limited situations. I have difficulty in or understanding extended conversations. I am unable to understand or communicate most healthcare concepts. (5)

Display This Question:

If Please select all the languages you speak and/or understand at any level, including English. = Portuguese

X→

© 2025 Hoffman H et al. *JAMA Network Open*.

Q104 Please select your level of fluency in Portuguese.

- **Native/Functionally Native:** I converse easily and accurately in all types of situations. Native speakers, including the highly educated, may think that I am a native speaker, too. (1)

- **Advanced:** I speak very accurately, and I understand other speakers very accurately. Native speakers have no problem understanding me, but they probably perceive that I am not a native speaker. (2)

- **Good:** I speak well enough to participate in most conversations. Native speakers notice some errors in my speech or my understanding, but my errors rarely cause misunderstanding. I have some difficulty communicating necessary health concepts. (3)

- **Fair:** I speak and understand well enough to have extended conversations about current events, work, family, or personal life. Native speakers notice many errors in my speech or my understanding. I have difficulty communicating about health care concepts (4)

- **Basic:** I speak the language imperfectly and only to a limited degree and in limited situations. I have difficulty in or understanding extended conversations. I am unable to understand or communicate most healthcare concepts. (5)

Display This Question:

If Please select all the languages you speak and/or understand at any level, including English. = Russian

X→

© 2025 Hoffman H et al. *JAMA Network Open*.

Q90 Please select your level of fluency in Russian.

◯ **Native/Functionally Native:** I converse easily and accurately in all types of situations. Native speakers, including the highly educated, may think that I am a native speaker, too. (1)

◯ **Advanced:** I speak very accurately, and I understand other speakers very accurately. Native speakers have no problem understanding me, but they probably perceive that I am not a native speaker. (2)

◯ **Good:** I speak well enough to participate in most conversations. Native speakers notice some errors in my speech or my understanding, but my errors rarely cause misunderstanding. I have some difficulty communicating necessary health concepts. (3)

◯ **Fair:** I speak and understand well enough to have extended conversations about current events, work, family, or personal life. Native speakers notice many errors in my speech or my understanding. I have difficulty communicating about health care concepts (4)

◯ **Basic:** I speak the language imperfectly and only to a limited degree and in limited situations. I have difficulty in or understanding extended conversations. I am unable to understand or communicate most healthcare concepts. (5)

Display This Question:

If Please select all the languages you speak and/or understand at any level, including English. = Spanish

© 2025 Hoffman H et al. *JAMA Network Open*.

Q91 Please select your level of fluency in Spanish.

◯ **Native/Functionally Native:** I converse easily and accurately in all types of situations. Native speakers, including the highly educated, may think that I am a native speaker, too. (1)

◯ **Advanced:** I speak very accurately, and I understand other speakers very accurately. Native speakers have no problem understanding me, but they probably perceive that I am not a native speaker. (2)

◯ **Good:** I speak well enough to participate in most conversations. Native speakers notice some errors in my speech or my understanding, but my errors rarely cause misunderstanding. I have some difficulty communicating necessary health concepts. (3)

◯ **Fair:** I speak and understand well enough to have extended conversations about current events, work, family, or personal life. Native speakers notice many errors in my speech or my understanding. I have difficulty communicating about health care concepts (4)

◯ **Basic:** I speak the language imperfectly and only to a limited degree and in limited situations. I have difficulty in or understanding extended conversations. I am unable to understand or communicate most healthcare concepts. (5)

Display This Question:

If Please select all the languages you speak and/or understand at any level, including English. = Swahili

X→

Q105 Please select your level of fluency in Swahili.

◯ **Native/Functionally Native:** I converse easily and accurately in all types of situations. Native speakers, including the highly educated, may think that I am a native speaker, too. (1)

◯ **Advanced:** I speak very accurately, and I understand other speakers very accurately. Native speakers have no problem understanding me, but they probably perceive that I am not a native speaker. (2)

◯ **Good:** I speak well enough to participate in most conversations. Native speakers notice some errors in my speech or my understanding, but my errors rarely cause misunderstanding. I have some difficulty communicating necessary health concepts. (3)

◯ **Fair:** I speak and understand well enough to have extended conversations about current events, work, family, or personal life. Native speakers notice many errors in my speech or my understanding. I have difficulty communicating about health care concepts (4)

◯ **Basic:** I speak the language imperfectly and only to a limited degree and in limited situations. I have difficulty in or understanding extended conversations. I am unable to understand or communicate most healthcare concepts. (5)

Display This Question:

If Please select all the languages you speak and/or understand at any level, including English. = Urdu

X→

© 2025 Hoffman H et al. *JAMA Network Open*.

Q106 Please select your level of fluency in Urdu.

- ◯ **Native/Functionally Native:** I converse easily and accurately in all types of situations. Native speakers, including the highly educated, may think that I am a native speaker, too. (1)

- ◯ **Advanced:** I speak very accurately, and I understand other speakers very accurately. Native speakers have no problem understanding me, but they probably perceive that I am not a native speaker. (2)

- ◯ **Good:** I speak well enough to participate in most conversations. Native speakers notice some errors in my speech or my understanding, but my errors rarely cause misunderstanding. I have some difficulty communicating necessary health concepts. (3)

- ◯ **Fair:** I speak and understand well enough to have extended conversations about current events, work, family, or personal life. Native speakers notice many errors in my speech or my understanding. I have difficulty communicating about health care concepts (4)

- ◯ **Basic:** I speak the language imperfectly and only to a limited degree and in limited situations. I have difficulty in or understanding extended conversations. I am unable to understand or communicate most healthcare concepts. (5)

Display This Question:

If Please select all the languages you speak and/or understand at any level, including English. = Uzbek

X→

© 2025 Hoffman H et al. *JAMA Network Open*.

Q107 Please select your level of fluency in Uzbek.

○ **Native/Functionally Native:** I converse easily and accurately in all types of situations. Native speakers, including the highly educated, may think that I am a native speaker, too. (1)

○ **Advanced:** I speak very accurately, and I understand other speakers very accurately. Native speakers have no problem understanding me, but they probably perceive that I am not a native speaker. (2)

○ **Good:** I speak well enough to participate in most conversations. Native speakers notice some errors in my speech or my understanding, but my errors rarely cause misunderstanding. I have some difficulty communicating necessary health concepts. (3)

○ **Fair:** I speak and understand well enough to have extended conversations about current events, work, family, or personal life. Native speakers notice many errors in my speech or my understanding. I have difficulty communicating about health care concepts (4)

○ **Basic:** I speak the language imperfectly and only to a limited degree and in limited situations. I have difficulty in or understanding extended conversations. I am unable to understand or communicate most healthcare concepts. (5)

Display This Question:

If Please select all the languages you speak and/or understand at any level, including English. = Vietnamese

X→

© 2025 Hoffman H et al. *JAMA Network Open*.

Q108 Please select your level of fluency in Vietnamese

○ **Native/Functionally Native:** I converse easily and accurately in all types of situations. Native speakers, including the highly educated, may think that I am a native speaker, too. (1)

○ **Advanced:** I speak very accurately, and I understand other speakers very accurately. Native speakers have no problem understanding me, but they probably perceive that I am not a native speaker. (2)

○ **Good:** I speak well enough to participate in most conversations. Native speakers notice some errors in my speech or my understanding, but my errors rarely cause misunderstanding. I have some difficulty communicating necessary health concepts. (3)

○ **Fair:** I speak and understand well enough to have extended conversations about current events, work, family, or personal life. Native speakers notice many errors in my speech or my understanding. I have difficulty communicating about health care concepts (4)

○ **Basic:** I speak the language imperfectly and only to a limited degree and in limited situations. I have difficulty in or understanding extended conversations. I am unable to understand or communicate most healthcare concepts. (5)

Display This Question:

If Please select all the languages you speak and/or understand at any level, including English. = Additional language 1 (please list only one language)

X→

© 2025 Hoffman H et al. *JAMA Network Open*.

Q92 Please select your level of fluency in your additional language (1).

◯ **Native/Functionally Native:** I converse easily and accurately in all types of situations. Native speakers, including the highly educated, may think that I am a native speaker, too. (1)

◯ **Advanced:** I speak very accurately, and I understand other speakers very accurately. Native speakers have no problem understanding me, but they probably perceive that I am not a native speaker. (2)

◯ **Good:** I speak well enough to participate in most conversations. Native speakers notice some errors in my speech or my understanding, but my errors rarely cause misunderstanding. I have some difficulty communicating necessary health concepts. (3)

◯ **Fair:** I speak and understand well enough to have extended conversations about current events, work, family, or personal life. Native speakers notice many errors in my speech or my understanding. I have difficulty communicating about health care concepts (4)

◯ **Basic:** I speak the language imperfectly and only to a limited degree and in limited situations. I have difficulty in or understanding extended conversations. I am unable to understand or communicate most healthcare concepts. (5)

Display This Question:

If Please select all the languages you speak and/or understand at any level, including English. = Additional language 2 (please list only one language)

X→

© 2025 Hoffman H et al. *JAMA Network Open*.

Q93 Please select your level of fluency in your additional language (2).

- ◯ **Native/Functionally Native:** I converse easily and accurately in all types of situations. Native speakers, including the highly educated, may think that I am a native speaker, too. (1)

- ◯ **Advanced:** I speak very accurately, and I understand other speakers very accurately. Native speakers have no problem understanding me, but they probably perceive that I am not a native speaker. (2)

- ◯ **Good:** I speak well enough to participate in most conversations. Native speakers notice some errors in my speech or my understanding, but my errors rarely cause misunderstanding. I have some difficulty communicating necessary health concepts. (3)

- ◯ **Fair:** I speak and understand well enough to have extended conversations about current events, work, family, or personal life. Native speakers notice many errors in my speech or my understanding. I have difficulty communicating about health care concepts (4)

- ◯ **Basic:** I speak the language imperfectly and only to a limited degree and in limited situations. I have difficulty in or understanding extended conversations. I am unable to understand or communicate most healthcare concepts. (5)

Display This Question:

If Please select all the languages you speak and/or understand at any level, including English. = Additional language 3 (please list only one language)

X→

© 2025 Hoffman H et al. *JAMA Network Open*.

Q94 Please select your level of fluency in your additional language (3).

- ○ **Native/Functionally Native:** I converse easily and accurately in all types of situations. Native speakers, including the highly educated, may think that I am a native speaker, too. (1)
- ○ **Advanced:** I speak very accurately, and I understand other speakers very accurately. Native speakers have no problem understanding me, but they probably perceive that I am not a native speaker. (2)
- ○ **Good:** I speak well enough to participate in most conversations. Native speakers notice some errors in my speech or my understanding, but my errors rarely cause misunderstanding. I have some difficulty communicating necessary health concepts. (3)
- ○ **Fair:** I speak and understand well enough to have extended conversations about current events, work, family, or personal life. Native speakers notice many errors in my speech or my understanding. I have difficulty communicating about health care concepts (4)
- ○ **Basic:** I speak the language imperfectly and only to a limited degree and in limited situations. I have difficulty in or understanding extended conversations. I am unable to understand or communicate most healthcare concepts. (5)

Page Break

© 2025 Hoffman H et al. *JAMA Network Open*.

Q6 Please answer the following to the best of your knowledge.

- ○ I was born in a country outside of the United States (first generation immigrant) (1)
- ○ One or both of my parents were born outside of the United States but I was born in the United States (second generation) (2)
- ○ One or multiple grandparents were born outside of the United States but my parents were both born in the United States (third generation) (3)
- ○ My grandparents, my parents, and I were all born in the United States (4)
- ○ I would describe my experience differently (please describe) (5) ____

End of Block: Demographics

Start of Block: Resources and Conference

Q82 Please continue to the next page to be redirected to a separate private survey to submit your information to receive a Target gift card to thank you for your participation in this study. Additional resources about including participants with limited English proficiency in your research are included in the next survey.

End of Block: Resources and Conference

© 2025 Hoffman H et al. *JAMA Network Open*.
