# Supplementary material for: Researcher Perceptions of Inclusion of Study Participants Who Use Languages Other Than English
Source: JAMA Netw Open. 2025 Mar 28;8(3):e252380. doi: 10.1001/jamanetworkopen.2025.2380 (PMC11953757; doi:10.1001/jamanetworkopen.2025.2380)
Supplement: Supplement 2. — Data Sharing Statement [file jamanetwopen-e252380-s002.pdf]

Data Sharing Statement

Hoffman. Researcher Perceptions of Inclusion of Study Participants Who Use Languages Other Than English. *JAMA Netw Open*. Published March 28, 2025.
doi:10.1001/jamanetworkopen.2025.2380

Data
Data available: Yes
Data types: Deidentified participant data, Data dictionary
How to access data: Can contact Maya Ragavan ragavanm@chp.edu to request data
When available: With publication

Supporting Documents
Document types: Informed consent form, Other (please specify)
Additional Information: Survey measures
How to access documents: Can contact Maya Ragavan ragavanm@chp.edu to request data
When available: With publication

Additional Information
Who can access the data: The data will be available to researchers whose proposed use of the data has been approved.
Types of analyses: For any purpose
Mechanisms of data availability: With a signed data agreement
